# Behavioural energetics in human locomotion: how energy use influences how we move

**DOI:** 10.1242/jeb.248125

**Published:** 2025-02-20

**Authors:** Megan J. McAllister, Anthony Chen, Jessica C. Selinger

**Affiliations:** School of Kinesiology and Health Studies, Queen's University, Kingston, ON K7L 3N6, Canada

**Keywords:** Locomotion, Gait, Metabolic cost, Energy minimization, Optimization, Biomechanics

## Abstract

Nearly a century of research has shown that humans, and other animals, tend to move in ways that minimize energy use. A growing body of evidence suggests that energetic cost is not only an outcome of our movement, but also plays a central role in continuously shaping it. This has led to an emerging research area, at the nexus between biomechanics and neuroscience, termed behavioural energetics, which is focused on understanding the mechanisms of energy optimization and how this shapes our coordination and behaviour. In this Review, we first summarize the existing evidence for and against our preferred locomotor behaviours coinciding with energy optima. Although evidence of our preference for energetically optimal gaits has existed for decades, new research is revealing its relevance across a surprising array of dynamic locomotor tasks and complex environments. We next discuss evidence that we adapt our gait toward energy optima over short timescales and in novel environments, which we view as a more stringent test that energy expenditure is optimized in real-time. This necessitates that we sense energy use, or proxies for it, on similar timescales. We therefore next provide an overview of candidate sensory mechanisms of energy expenditure. Finally, we discuss how behavioural energetics can be applied to novel wearable assistive technologies and rehabilitation paradigms, and conclude the Review by outlining what we see as the most important future challenges and opportunities in behavioural energetics.

## Teaser

**Summary:** In this Review, we focus on historical and emerging research investigating the principle of energy optimization and how it shapes human locomotor mechanics and behaviour.

## Introduction

Although human biomechanists have traditionally studied the mechanical determinants of energetic cost, there is a growing interest in the inverse of this relationship – how our energy requirements determine how we move. For example, decades of fundamental biomechanical research has disentangled the energetic costs associated with various aspects of human gait, such as body weight support, propulsion and leg swing (Arellano and Kram, 2011; Arellano and Kram, 2014b; Donelan et al., 2004; Gottschall and Kram, 2003, 2005; Grabowski et al., 2005; Kipp et al., 2018; Kram and Taylor, 1990; Peyre-Tartaruga et al., 2021). These works largely frame energetic cost as the result of our mechanics. However, a growing body of evidence suggests that energetic cost is not only an outcome of our movement, but also plays a central role in continuously shaping it. This research area, bridging biomechanics and neuroscience, has been referred to as behavioural energetics (Selinger and Donelan, 2019). Here, we define it as a focus on ‘understanding the mechanisms of energy optimization and how this shapes our coordination and behaviour’.

Nearly a century of research has shown that humans, and other animals, tend to move in ways that minimize energy use (Alexander, 1996; Atzler and Herbst, 1928; Elftman, 1966; Molen et al., 1972; Ralston, 1958; Zarrugh et al., 1974). For example, humans typically walk at a speed that minimizes their cost of transport (calories expended per unit distance travelled) (Molen et al., 1972; Ralston, 1958; Zarrugh et al., 1974). At that speed, they also select other gait parameters, such as step frequency or width, that are also energetically optimal (Bertram and Ruina, 2001; Donelan et al., 2001; Holt et al., 1991; Minetti et al., 1995; Umberger and Martin, 2007). Although beyond the scope of this review, preferences for energy optimal locomotor behaviours have been demonstrated in other non-human animals, be it galloping horses, flying birds and swimming fish (Hoyt and Taylor, 1981; Li et al., 2020; Weimerskirch et al., 2001). That our preferred gaits coincide with energy minima suggests energy optimization is a fundamental principle of motor control but does not make clear over what timescale this optimization has occurred or is possible. There is evidence that the energy optimality of our gait is partially established over evolutionary timescales, through changes in body morphology and hardwired neural circuitry (Alexander, 1996, 2001; Rodman and McHenry, 1980; Sockol et al., 2007). It is also possible that, over our lifetime, our preferred gaits simply become energy optimal given years of practice that subtly tune our physiology and energy economy (DeJaeger et al., 2001; Ivanenko et al., 2007). However, adaptations over these timescales cannot satisfactorily explain energy-minimizing behaviours that occur in novel contexts over short timescales. Behavioural energetics focuses on an additional possibility: that energetic cost is continuously optimized, in real-time, to guide our movements. There is growing evidence that the principle of energy minimization may explain a surprising number of behaviours beyond steady-state healthy walking, including how we learn new motor coordination patterns, move intermittently and navigate obstacles (Brown et al., 2021; Daniels and Burn, 2023; Darici and Kuo, 2022; Long and Srinivasan, 2013).

Although energy minimization appears to be a primary motor control objective, it is certainly not the only objective, and its relative importance undoubtedly changes with context and task. Several other important factors, such as speed (time), stability, agility and avoidance of discomfort/pain (Bornstein and Bornstein, 1976; Carlisle and Kuo, 2023; Hunter et al., 2010; McDonald et al., 2022, 2019; Price et al., 2023), either in combination with or in addition to energetic cost, also influence our preferred movements. How we weigh each of these factors is likely affected by the movement task, environment and constraints on our body. However, when there is flexibility in how these other objectives are met, people appear to prefer energy optimal solutions. For example, you may walk faster to catch a departing bus, but within this speed constraint you still walk at an energetically optimal step frequency. Behavioural energetics is in its infancy, given how little we currently know about energy trade-offs with other competing objectives across different contexts. Studying when and how humans depart from energy optimal behaviour can provide insight into the relative importance of other objectives.

Real-time energy optimization, particularly in complex, changing or novel contexts, necessitates that the nervous system senses, integrates and re-evaluates energy expenditure along with other sensory signals. This, at its core, is a learning problem – and a challenging one. To allow for rapid adaptation, the nervous system must sense (or predict) energy expenditure (or some proxy for it) with minimal latency, but we know relatively little about how this is accomplished. Even with perfect sensing, the efferent nervous system has tens of thousands of motor units at its disposal, the firing rate of which can be adapted many times per second. This results in near countless degrees of freedom in possible motor coordination patterns that cannot be exhaustively explored over short timescales (Bellman, 1952; Bernstein, 1966). How the nervous system searches the expanse of possible coordination strategies remains an open and important question. Considering this, we view energy optimization in novel tasks as a learning process – one that may rely on approximation of the actual cost function, takes time and requires experience. This means that the nervous system may not immediately or perfectly converge on optima. Carefully designed experiments have revealed energetically irrational behaviours, even in situations where energy minimization appears to be an objective of the nervous system. These ‘mistakes’ may provide unique insight into sensing mechanisms and the limits of the explanatory power of energy optimization.

In this Review, we summarize historical and recent works in human behavioural energetics, and identify future opportunities and challenges in this emerging field. We first summarize the existing evidence for and against our preferred behaviours coinciding with energy optima. We next summarize evidence that our behaviours are adapted toward energy optima over short timescales. Here, we separate evidence into that which shows changes in energy cost track motor adaptation and drive motor adaptation, the latter of which we view as a more stringent test of real-time energy optimization. Next, we review candidate sensory mechanisms of energy expenditure, summarizing the existing evidence and outlining the theoretical advantages and disadvantages for each. We then discuss how behavioural energetics can be applied to novel wearable assistive technologies and rehabilitation paradigms. We conclude the Review by outlining what we see as the most important future directions in behavioural energetics. We have limited the scope of this Review to human locomotion, although we note that some have explored the principle of energy optimization in non-locomotor tasks, such as upper arm reaching (Huang et al., 2012; Kistemaker et al., 2010; Lyons et al., 2006; Wong et al., 2021).

## Evidence that preferred behaviours coincide with energy optima

Fundamental characteristics of our gait align with energy minima. While the research we summarize here cannot disentangle whether energy minimizing gait characteristics are largely established over millennia, years or minutes, they nonetheless provide some of the most concrete and long-standing evidence that our preferred gaits minimize energy. The experimental approach used in many of these early studies is a simple one: to investigate the energy optimality of a given gait characteristic, a person walks at their preferred gait and is then directed to alter a gait characteristic of interest to higher and lower values, often using visual or auditory feedback. Each unique gait bout usually lasts five or more minutes to allow for steady-state measurements of metabolic energy expenditure from indirect calorimetry (Brockway, 1987; Poole and Richardson, 1997; Selinger and Donelan, 2014). The resulting relationship between the gait characteristic of interest and metabolic energy expenditure is termed the cost landscape or cost curve. In the sections below we present seminal experiments from walking, running and cycling, all largely conducted in steady-state laboratory conditions. We then review more recent works examining locomotion during non-steady-state and free-living settings, where tasks are more complex and competing objectives are likely.

### Steady-state locomotion

#### Walking

**Fig. 1.**
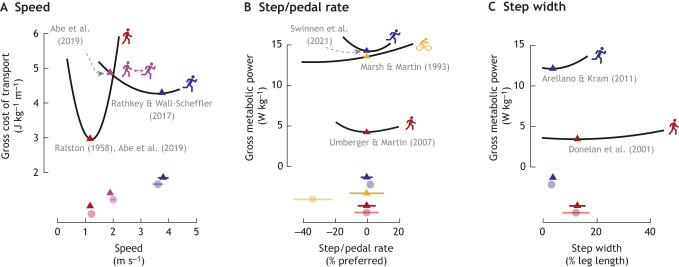
**Preferred gait characteristics and energy optima often coincide in steady-state locomotion.** Cost landscapes for locomotor speed (A), step/pedal rate (B) and step width (C) for walking (red), running (blue), walk–run transition (purple) and cycling (yellow). Triangles illustrate preferred values and circles illustrate energy optimal values (minima). Horizontal lines represent ±1 s.d. and are presented when available. See Supplementary Materials and Methods for additional figure generation details.

Spatiotemporal gait characteristics tend to align with energy minima during steady-state, level walking. Ralston (1958) was among the first to demonstrate this for walking speed, showing that preferred speeds minimize the gross cost of transport (often normalized to body weight and measured in units of J m^−1^ kg^−1^ or cal m^−1^ kg^−1^) (Fig. 1A). Notably, the speed that minimizes the gross cost of transport is higher than the speed that minimizes net cost of transport, where the energy expenditure associated with basal metabolic rate (often assessed during standing, sitting or lying) is removed (Brooks et al., 2005; Glass et al., 2007). Most studies have replicated Ralston's findings, showing that preferred speeds align with gross cost of transport (Baroudi et al., 2024; Browning and Kram, 2005; Peyrot et al., 2012; Wickler et al., 2001), although others have shown alignment to net cost of transport (Courter et al., 2023; Gidley and Lankford, 2021; Rock et al., 2018) and some recent evidence suggests that, in free-living settings, speeds better align with net cost of transport (Selinger et al., 2022). This perhaps aligns well with Srinivasan's argument that minimizing the net cost of transport is akin to maximizing the distance travelled on a fixed amount of energy, whereas minimizing gross cost of transport is akin to minimizing the total daily energy consumption (Srinivasan, 2009). Srinivasan argues that the former is energetically rational in contexts where food is scarce and long distances must be travelled to secure it, and the latter is more energetically rational in contexts of many modern lifestyles, where that is not the case and sedentary times are higher. However, because there is no consensus (with some reporting that preferred speeds match the gross and others the net cost of transport), this limits the certainty we can have in these findings. When two possible formulations of the ‘optimal cost’ exist and measures are inherently noisy, it can create a relatively large range of preferred speeds that could be deemed ‘optimal’. Ideally, experiments would be powered to statistically rule out gross versus net optima (Selinger et al., 2022); however, this can require a prohibitively large number of participants. Other preferred spatiotemporal gait characteristics, such as step frequency, step length or step width, have also been found to minimize metabolic power (Bertram and Ruina, 2001; Donelan et al., 2001; Holt et al., 1991; Minetti et al., 1995; Umberger and Martin, 2007) (Fig. 1B,C). Here, when speed is fixed, energy expenditure is assessed per unit time, not distance, in units of power (W kg^−1^). This means that net and gross minima do not differ. Much of this experimental evidence is supported by insights from simple dynamic walking models that can reveal underlying trade-offs that lead to energy optima (Kuo, 2001, 2007; Kuo and Donelan, 2010). For example, the energy optimal step frequency can be understood as a trade-off between step-to-step transition costs, where leg collision losses require positive push-off work that decreases with faster step rates, and leg swing costs that increase with faster step rates (Donelan et al., 2002; Kuo, 2007). Decades of laboratory experiments and theoretical modelling have collectively demonstrated that in natural, steady-state, level walking conditions, our gaits are energy optimal.

When terrains are altered or tasks are constrained, there is clear evidence that people will forgo energy optimal gaits in some contexts. For example, Hunter et al. (2010) showed that in decline walking, instructing participants to walk in a ‘relaxed manner’ can decrease muscle activity and whole-body energy expenditure. Accompanying dynamic walking models predicted reduced actuation requirements during downhill walking and decreased stability (Hunter et al., 2010). Together, this suggests people do not fully exploit the propulsion provided by gravity and instead prefer a more stable, but costlier, gait. This finding is unique to decline walking – there is no known instruction that can be given during healthy level walking to reduce costs. Another example is a clever experiment by McDonald et al. (2022), which demonstrated that when explicitly forced to choose between a crouch gait causing high ‘fatigue-like’ muscle activation or an incline gait leading to higher metabolic cost, participants chose the latter. This suggests that factors such as local muscle fatigue or unfamiliar coordination patterns may cause people to prefer energetically suboptimal solutions. Alternatively, a muscle activation-based control signal may be sensed and optimized instead of, or as a sometimes-flawed proxy for, whole-body metabolic cost (discussed further in the section ‘Candidate sensory mechanisms of energy use’). These departures from energy optimal gaits offer insights into the importance of other objectives, such as stability or avoidance of discomfort, in each context. Methods for systematically assessing and quantifying the relative importance of key gait objectives – energy, stability, speed/time, agility and pain/discomfort – across varying tasks, terrains and individuals would greatly benefit the field.

#### Running

Since Margaria et al.’s early experiments published in 1963, human runners were thought to consume a constant amount of energy for a given distance travelled, regardless of running speed (Carrier et al., 1984; Margaria et al., 1963; Menier and Pugh, 1968). That is, although the cost of running per unit time increases with increasing speed (for a given amount of time, it is costlier to run faster), the cost of running per unit distance was thought to be near independent of speed (for a given distance, one was thought to burn a similar number of calories whether running fast or slow). This trait has been hypothesized to confer a competitive advantage to our early ancestors during long-distance hunting by allowing them to adapt their running speed to be least economical for their prey, with minimal energetic consequence (Bramble and Lieberman, 2004; Carrier et al., 1984). However, more recent experiments by Wall-Scheffler and others more carefully measured energetics across a range of speeds and repeats on a treadmill, and found that while the gross cost of transport curve is shallower in running than walking, there is nonetheless an energetically optimal running speed that corresponds to runners' preferred speed (Rathkey and Wall-Scheffler, 2017; Steudel-Numbers and Wall-Scheffler, 2009; Willcockson and Wall-Scheffler, 2012) (Fig. 1A). This optimum appears to correspond to runners' preferred speed when asked to select a pace they could comfortably sustain for 1 h, for example when they are out for a jog and not a race (Rathkey and Wall-Scheffler, 2017). This is distinct from a runner's performance capability (i.e. competitive race times), where minimizing time is the explicit goal. Interestingly, some evidence suggests that an energy optimal running speed (be it gross or net) is more evident in experienced runners, who more consistently modulate cadence with speed and tend to have higher aerobic capacities (Cher et al., 2015). This suggests that optima may be developed or refined with training, although to our knowledge no study has directly tested this. Numerous experiments have demonstrated that runners also select other gait parameters, such as step frequency (Gutmann et al., 2006; Snyder et al., 2012; Swinnen et al., 2021), step length (Gutmann et al., 2006), step width (Arellano and Kram, 2011) and arm swing (Arellano and Kram, 2014a), to minimize metabolic cost across level, incline and decline conditions (Sheehan and Gottschall, 2013; Snyder and Farley, 2011) (Fig. 1B,C).

#### Cycling

Unlike walking and running, cycling is a form of locomotion where preferred coordination patterns do not align well with energy minima. For example, Marsh and Martin (1993) found that the most economical cycling cadence (an analogous metric to step frequency) was roughly 50% lower than preferred cadences; however, this large difference results in only ∼5% elevated costs (Fig. 1B). Here, participants were pedalling on a stationary bicycle at 200 W, and preferred cadence was that which participants selected when instructed to pedal at a pace that would be comfortable for an extended period (>1 h). Formenti et al. (2019) more directly measured the degree of oxygenation (tissue saturation index) in the vastus lateralis during cycling across a range of cadences using near-infrared spectroscopy (NIRS). However, a clear relationship between preferred cycling cadence and the degree of oxygenation was not found (Formenti et al., 2019; Shastri et al., 2019). Others have investigated what distinct objectives might explain these preferences, including minimizing lower extremity net joint moments (Marsh et al., 2000) and applied pedal forces (Sanderson, 1991). Muscle activation-based metrics are also a leading hypothesized objective (Brennan et al., 2019; Riveros-Matthey et al., 2023 preprint). In human cycling experiments, optimal cadences for summed muscle activation metrics tend to be indistinguishable from net metabolic power (Brennan et al., 2019). However, recent work by Riveros-Matthey et al. (2023) used musculoskeletal modelling to dissociate the two and showed that preferred cadences more closely aligned with simulated averaged muscle activation volume than energy expenditure. Given that cycling kinematics are highly constrained (limiting the ability to make compensatory changes to muscle activation), avoiding individual muscle fatigue may be a particularly important priority, as higher cadences do appear to affect muscle oxygenation near the ventilatory threshold (Formenti et al., 2019; Shastri et al., 2019). Or, given that cycling is a relatively new form of locomotion, and not one we have evolved for, it is possible that activation is a flawed proxy for energy use in this particular context. However, over longer durations, preferred and energy optimal cadences do begin to converge (Brisswalter et al., 2000), suggesting the nervous system may be slowly optimizing for cost or upweighting the relative importance of energy over longer rides.

### Non-steady-state locomotion

Recent studies have begun to explore the principle of energy optimization in more free-living contexts – during non-steady-state locomotion and across complex or changing terrains. This is important given that steady-state laboratory conditions may bias findings toward energy-based objectives and do not accurately reflect daily locomotion, where we string together many short bouts of steps between rests or non-walking behaviours (Orendurff et al., 2008). Transient costs associated with changing speeds could account for 4–8% of our daily energy budget (Seethapathi and Srinivasan, 2015). If energy optimization is a general movement principle, it should have explanatory power during more complex free-living tasks. However, directly and accurately measuring metabolic energy expenditure during dynamic movements is difficult (Ingraham et al., 2019; Selinger and Donelan, 2014). A necessary limitation of many of the studies discussed below is that optimal control models, in combination with available steady-state metabolic cost data, are used to predict time-varying energetically optimal behaviours.

#### Walk–run transitions, mixtures and changing speeds

There exists a particular speed at which it becomes metabolically more expensive to walk than to run: the walk–run transition speed (typically around 2 m s^−1^ or 0.5 Froude number) (Diedrich and Warren, 1995; Hreljac, 1993). Humans will switch from a walking to running gait near this speed; however, several groups have scrutinized whether that switch is metabolically driven (Mercier et al., 1994; Minetti et al., 1994; Raynor et al., 2002; Rotstein et al., 2005). Most studies report that participants transition to a run at a speed ∼5% lower than the speed that minimizes gross cost of transport when the treadmill speed is gradually increased (Abe et al., 2019; Rotstein et al., 2005; Ziv and Rotstein, 2009). However, this lower transition speed appears sensitive to the treadmill speeding up (walk–run) or slowing down (run–walk), equates to only a 1–2% increase in energy cost and is not reproduced during overground locomotion, suggesting it may be an artifact of the experimental paradigm (Abe et al., 2019; Van Caekenberghe et al., 2010; Ziv and Rotstein, 2009). An extensive review of other proposed objectives to explain the walk–run transition point can be found in Kung et al. (2018).

**Fig. 2.**
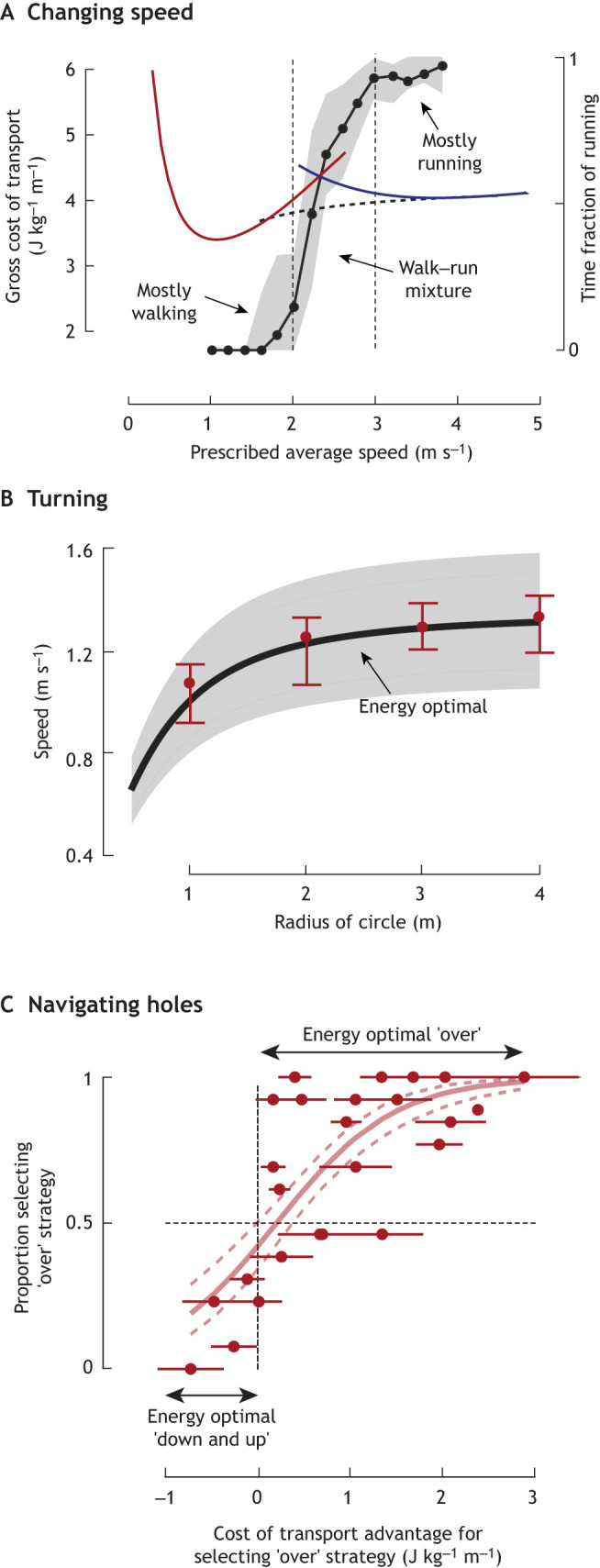
**Humans exhibit energy optimal behaviours in non-steady-state locomotion.** (A) A mixture of walking and running gaits are energy optimal near the intersection of the walk (red) and run (blue) cost landscapes, given the non-convexity of the effective cost curve (dashed curve). Participants self-select these walk–run mixtures (black line, grey shading is 25th to 75th percentile) when in the transition region between 2 and 3 m s^−1^ (vertical dashed lines), while selecting mostly walking and mostly running at lower and higher speeds, respectively. Data from Long and Srinivasan (2013). (B) When navigating turns of various radii, people select speeds that are predicted by energy optimality (black line, grey shading is ±2% of optimal energy cost). Red circles are across participant median speeds and error bars indicate the 25th to 75th percentile. Data from Brown et al. (2021)*.* (C) When navigating cuboidal holes in the ground (of lengths 0.5–1.1 times leg length and depths of 0.1–0.5 times leg length), people select a strategy – stepping ‘over’ or ‘down and up’ – that is predicted by energy optimality. Red circles are across participant mean proportion for each length-depth combination (±1 s.d. as horizontal lines). Solid shaded red line represents the fitted logistic curve and dashed lines represent 95% confidence interval. Data from Daniels and Burn (2023). See Supplementary Materials and Methods for additional figure generation details.

In more ecological settings, people appear to select walk–run–rest mixtures and dynamically change their speed in accordance with the principle of energy minimization. Long and Srinivasan (2013) asked participants to travel a given distance overground and prescribed the amount of time they had to do so, thus constraining their average speed. As expected, participants walked the entire distance when given ample time and mostly ran when time was limited. Importantly, at intermediate speeds, participants used a mixture of walking and running bouts, as opposed to choosing one constant locomotor speed. Using existing cost of transport data and computational optimization, the authors demonstrated that the effective cost curve (one generated by selecting the lowest expenditure gait at any given speed) is non-convex at the intersection of the walk and run curves (Long and Srinivasan, 2013). This non-convexity, by definition, means that the lowest attainable average energy rate is only realized using a mixture of walking and running gaits near the intersection speed (as opposed to exclusively walking or running) – precisely as participants do (Fig. 2A). This formulation holds even when transient costs (the energy cost associated with accelerating or decelerating between gaits) are considered (Long and Srinivasan, 2013; Seethapathi and Srinivasan, 2015). However, there are locomotor behaviours not explainable by the Long and Srinivasan (2013) model. When switching between rest and walking (or vice versa), people gradually increase (or decrease) their speed, as opposed to immediately walking at the optimal steady-state speed (Carlisle and Kuo, 2023). This results in a speed versus time trajectory that is an inverted U-shape. Carlisle and Kuo (2023) explained this behaviour using a computational walking model that included a cost proportional to time. By optimizing an objective function that includes both energy and time, their model can explain individual- or context-dependent vigour when selecting speed (how much one values time relative to energy). This may explain why some people tend to walk faster than others (Labaune et al., 2020), why city size affects pedestrian walking speed (Bornstein and Bornstein, 1976; Levine and Bartlett, 2016) and why we are willing to rush to catch that departing bus.

#### Obstacle avoidance and complex terrains

There is new evidence that the locomotor speeds and paths we choose during non-straight-line walking – when turning and avoiding obstacles – can also be explained by energy optimality. Brown et al. (2021) measured net metabolic cost while participants walked overground in circles of varying radii at constrained speeds, allowing them to derive a turning radius versus walking speed cost landscape. Using these data, they mathematically predicted energy optimal behaviours across a range of complex path trajectories (Fig. 2B). Their computations can explain a surprising range of naturalistic human walking behaviours (many of which one might have assumed were governed by other objectives such as stability). For example, energy optimality can explain why we turn slowly and avoid sharp turns, as well as how we navigate around obstacles and through corridors or doorways. They counterintuitively showed that the shortest path between two points is not always energy optimal and people will walk longer distances to save energy (Brown et al., 2021). There is also new evidence that, when confronted with uneven and irregular terrain surfaces, anticipatory adjustments to gait can be explained by energy optimality. When choosing between stepping across a hole in the ground or down and back up, people prefer the energy rational strategy across a wide range of hole depths and lengths (Daniels and Burn, 2023) (Fig. 2C). Similarly, when stepping across uneven terrain, people make anticipatory speed adjustments that are consistent with minimizing energy expenditure (Darici and Kuo, 2022, 2023). Planning in advance of complex terrain and making these anticipatory step adjustments necessitates the use of vision to predict an energetically rational locomotor strategy.

#### Free-living locomotion

Advances in wearable technology have created new opportunities to understand the relevance of behavioural energetics in more complex free-living settings. Some groups have partnered experimental measures of energetics with free-living observations of behaviour from global positioning system (GPS) and inertial measurement unit (IMU) data to leverage the benefits of each approach (Baroudi et al., 2024; Selinger et al., 2022). For example, using a commercial database of thousands of recreational runners, Selinger, along with Delp and colleagues, showed that preferred speed is largely unaffected by the distance run and is consistent with the speed that minimizes the net cost of transport (Selinger et al., 2022). Baroudi et al. (2024) used a similar approach in walking to show that preferred speed is often energy optimal (aligns with gross cost of transport) but is influenced by walking context. For example, participants on average walked faster if commuting compared with other contexts (behaviour likely captured by an objective function that includes both energy and time to account for urgency; Carlisle and Kuo, 2023). Technologies and methods to estimate energy expenditure from wearable devices during dynamically changing movements in free-living settings have also improved significantly (Ingraham et al., 2019; Mohammad et al., 2023; Slade et al., 2021). As these approaches and technologies gain wider acceptance and validation, they will provide new insights about the relationships not only between energetics and mechanics, but also task demands, built environments and social contexts.

## Evidence that behaviours are adapted toward energy optima

Alignment between preferred and energy optimal behaviours, as evidenced in the previous section, could occur over lifetime or evolutionary timescales for commonly encountered contexts. Here, we explore evidence suggesting that energetically favourable preferences can adapt over short timescales, on the order of minutes, even in novel environments that contradict past experiences. We consider this a more stringent test of the energy optimization hypothesis and evidence that energy may be sensed and optimized by the body in real-time.

### Evidence that changes in energy cost track gait adaptations

In novel locomotor contexts, reductions in energy expenditure have been shown to track kinematic adaptations over time. For example, in split-belt treadmill walking, where the belt under one limb moves faster than the other, walkers change step lengths over time to reduce asymmetries (Reisman et al., 2005). This has long been framed as minimization of sensory prediction error, where adaptation toward symmetrical steps reduces discrepancies between expected and actual sensory consequences (Choi et al., 2009; Morton and Bastian, 2006). Finley et al. (2013) were the first to demonstrate that reductions in metabolic cost track motor adaptations toward symmetry, suggesting that economy may be driving these changes in coordination. This adaptation involves learning to use the positive work produced by the split-belt treadmill, thereby reducing the required biological power and resulting metabolic energy expenditure (Sanchez et al., 2019), although the adapted step lengths appear to coincide with an optimal solution that combines minimizing energy and mechanical costs (Sanchez et al., 2017). That a split-belt treadmill can provide external assistance makes it analogous to more obvious assistive devices, such as lower-limb exoskeletons. Growing evidence suggests that, with time and experience, humans donning exoskeletons adapt their motor coordination to reduce metabolic energy expenditure (Abram et al., 2022; Ferris et al., 2007; Poggensee and Collins, 2021; Sawicki et al., 2020; Zhang et al., 2017). However, in all these experiments, causality is difficult to establish. Are kinematic or kinetic metrics being optimized and is metabolic energy coincidently lowered? Or, is metabolic energy being directly optimized and are kinematic or kinetic adaptations a means to that end? Moreover, without an unambiguous energetically optimal solution, it is unclear whether these behaviours reflect true energy optimization or are merely adaptations towards habitual behaviours that are satisfactory solutions (de Rugy et al., 2012; Loeb, 2012).

### Evidence that changes in energy cost drive gait adaptations

**Fig. 3.**
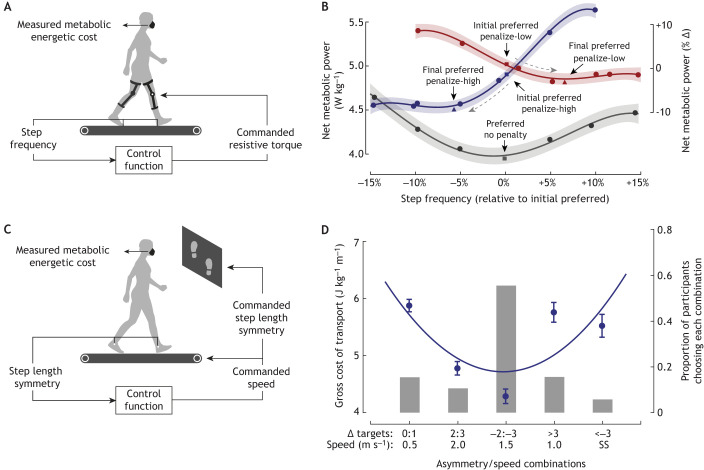
**Altering energetic consequences can drive gait adaptation.** (A) Experimental paradigm in which exoskeletons are used to apply a resistance to the limb that is proportional to the participants' step frequency, altering the energy optimal step frequency. (B) Resulting cost landscapes with optima shifted to a higher (red, penalize-low controller) or lower (blue, penalize-high controller) step frequency, compared to natural walking (grey, controller off). Circles indicate across participant averages. The lines are fourth-order polynomial fits for illustrative purposes, and the shading shows their 95% confidence intervals. (C) Experimental paradigm where step length asymmetry was coupled to treadmill speed, altering the energy optimal step symmetry/speed combination. (D) Resulting cost landscape with optimum shifted to an asymmetric gait. The blue circles indicate across participant average, error bars are 1 s.e.m. The blue line is a second-order polynomial fit for illustrative purposes. Grey vertical bars represent the proportion of participants choosing each asymmetry/speed combination. Adapted, with permission, from Selinger et al., 2015 (A,B) and based on Roemmich et al., 2019 (C,D). See Supplementary Materials and Methods for additional figure generation details.

Experimental paradigms that intentionally manipulate the energetic consequences of movement provide a stronger test of the energy optimization hypothesis. Our group, along with others, have developed real-time closed-loop mechatronic systems to intentionally shift the cost landscape minima, directly testing whether energy optimization drives locomotor adaptations, rather than simply tracks them. In an early experiment, Selinger, along with Donelan and colleagues, used an actuated knee exoskeleton to make resistive torques proportional to participants' step frequency, shifting the energy optimal step frequency either higher or lower (Selinger et al., 2015, 2019) (Fig. 3A). The authors showed that following adequate experience, participants adapted to the novel optima – discovering, adopting and learning to predict the new gaits – even when the energetic benefits were small (Fig. 3B). Using a similar closed-loop manipulation, Roemmich et al. (2019) coupled asymmetric stepping and treadmill gait speed, creating a situation where the preferred step length symmetry was no longer energetically optimal (Fig. 3C). They, too, demonstrated that people altered their preferences toward the novel energy optima, forgoing step length symmetry (Roemmich et al., 2019) (Fig. 3D). The Donelan group, along with Selinger, have since reproduced the original findings using a distinct paradigm that manipulated optimal step width (Abram et al., 2019). However, less clear evidence has been found when their paradigm is applied to overground walking where speed and terrain vary (Wong et al., 2019), as well as when landscapes are more dramatically altered using forces at the waist (Simha et al., 2021). This may be due to competing priorities, such as stability (McDonald et al., 2019), fatigability (McDonald et al., 2022) and/or discomfort (Yandell and Zelik, 2016), as well as a more challenging credit assignment problem (where ascribing costs to particular muscles or gait characteristics may be more difficult; Minsky, 1961; Sutton and Barto, 2018; Wolpert et al., 2011). Collectively, approaches that manipulate cost landscapes in real-time offer a more stringent test of the energy minimization hypothesis and a paradigm to interrogate the mechanisms that underlie it.

In these novel contexts, where energetic consequences are manipulated, the nervous system must search for new optimal coordination patterns. We have previously framed this as a reinforcement learning problem – where the nervous system could continually search (explore) for more optimal gaits or could simply remain at (exploit) a gait that is expected to be optimal (Selinger et al., 2019). Movement variability in learned and novel contexts can offer insight into the mechanisms of energy minimization. For example, Selinger, along with Donelan and colleagues, found that participants with naturally high step-to-step variability (explorers) are more likely to spontaneously adapt their gait to novel optima. Those with lower variability (exploiters) can be encouraged to begin optimization if provided with the experience of a lower cost gait (Selinger et al., 2015, 2019). Relatedly, Abram et al. (2022) found that when learning to walk with assistive lower limb exoskeletons, gait variability increased upon initial exposure to the new context and then decreased with experience. They suggest this general reduction in variability, which was correlated with decreased energy expenditure, represents a reduction in the search space as the nervous system adapts toward a new optimal gait. Poggensee and Collins (2021) also demonstrated that exposure to varied exoskeleton torque profiles (external variability) benefited learning. While these studies offer insight into the mechanisms that may underlie real-time energy optimization, much remains to be understood.

## Candidate sensory mechanisms of energy use

**Fig. 4.**
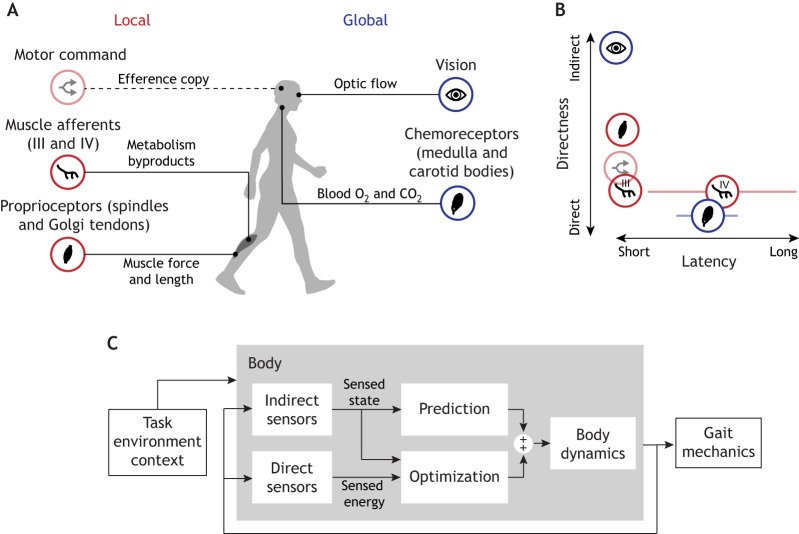
**Candidate sensory mechanisms of energy use.** (A) Possible local (red) and global (blue) sensors that indirectly or directly estimate energy use. Note that an efference copy (dashed line), derived from a motor command, is not a feedback sensory mechanism, but could be used to generate a feed-forward prediction about the energetic consequences of movement. (B) Theoretical plot of each sensor's response latency versus directness. (C) Conceptual diagram illustrating how behavioural energetics may involve indirect sensors that inform fast prediction and direct sensors that inform a slower optimization process to alter preferred gait mechanics. Adapted, with permission, from O'Connor and Donelan (2012). See Supplementary Materials and Methods for additional figure generation details.

If humans adapt their movements, over short timescales and in novel contexts, to reduce energy expenditure, the nervous system must be capable of sensing energy (Dean, 2013). However, little is known about what physiological sensors may provide a measure or estimate of energy, what sensorimotor pathways and projections are involved, and how various energetic sensory signals are centrally weighted and integrated over time. Here, we summarize the evidence for various hypothesized sensory pathways (Fig. 4), emphasize the theoretical advantages and disadvantages of each, and highlight existing knowledge gaps.

### Blood gas chemoreceptors

Blood gas chemoreceptors, which provide direct information regarding the internal metabolic and chemical state within the bloodstream, are logical candidates for sensing energy use. These sensors, located at the brainstem medulla oblongata and the aortic and carotid bodies, are sensitive to blood oxygen (O_2_) and carbon dioxide (CO_2_) (substrates and byproducts of oxidative metabolism, respectively) (Casaburi et al., 1977; Heymans, 1927; Whipp and Ward, 1998), and could therefore provide a ‘global’ (whole-body) measure of energy use. Moreover, these sensors have been shown to directly affect respiration during exercise. At the onset of exercise, when the energy demands of the body increase, ventilation rate rapidly increases, leading to greater O_2_ concentration in the bloodstream, which facilitates muscle cells in consuming more energy through cellular oxidative metabolism. Blood gas chemoreceptors are thought to contribute to ventilatory control because their afferent feedback precedes changes in ventilation (Casaburi et al., 1977; Forster et al., 1993; Whipp and Ward, 1998) and surgical removal of these sensors blunts the ventilatory response to exercise (Lugliani et al., 1971; Miller and Tenney, 1975; Wasserman et al., 1975). Given that chemoreceptors directly sense substrates and byproducts of metabolism, and appear to regulate physiological processes in response to changing energy demands, they are logical sensors of the body's total energy use. This may also suggest that blood gas chemoreceptors could be sensitive to a measure more akin to gross cost, whereas other more local, muscle-level sensors may reflect net cost. However, the response from these sensors can have a significant latency of ∼10–20 s (Dahan et al., 1990; Smith et al., 2006), owing to the time taken for the change in peripheral blood gas concentrations to reach chemoreceptors (∼10 s; Band et al., 1980) and the time taken for a change in blood concentrations to elicit an afferent response (∼1–6 s; Black et al., 1971; Fitzgerald et al., 1969). To test whether these chemoreceptors are used to sense and optimize energy expenditure during walking, Wong et al. (2017) used a custom end-tidal forcing gas control system developed by O'Connor et al. (2016) to manipulate breath-by-breath inspired gas concentrations (a proxy for arterial blood gas concentrations). They did so as a function of step frequency, creating a simulated energetic minimum (with high O_2_ and low CO_2_ concentrations) away from the naturally preferred step frequency. Importantly, the actual energetic cost of walking was left unchanged. Despite this manipulation causing a substantially higher ventilation rate and perceived exertion, participants continued at their normally preferred step frequency, suggesting that blood gas chemoreceptors play a negligible role in sensing energy use. However, it is possible that the manipulated chemoreceptors were downweighted by the nervous system in favour of other unperturbed energy-sensing pathways. It may also be that a global measure of energy expenditure in isolation, without supporting and non-contradictory signals from muscles, creates a unique challenge, as little information about how to adapt is available. Testing whether blood gas chemoreceptor manipulations can disrupt adaptation toward energy minimum in established paradigms (Abram et al., 2019; Roemmich et al., 2019; Selinger et al., 2015) could provide additional insight.

### Group III/IV metabosensitive muscle afferents

Some group III and IV muscle afferents are sensitive to metabolism byproducts, making them another logical candidate for sensing energy use. These muscle afferents respond to the accumulation of lactic acid, potassium ions and hydrogen ions in the muscle (Rotto and Kaufman, 1988; Rotto et al., 1989; Rybicki et al., 1985), and in turn contribute to the exercise pressor reflex (Amann et al., 2010; Kaufman and Hayes, 2002). This reflex triggers muscle sympathetic nerve activity to increase ventilatory and circulatory responses during exercise, and can signal muscle fatiguability (Gandevia, 2001). Blunted cardiovascular response occurs during blood flow occlusion, leading to metabolite accumulation (Adreani and Kaufman, 1998; Crisafulli et al., 2011), or when central projections of group III and IV muscle afferents are anesthetized (Amann et al., 2010, 2011). Blood flow restriction at the leg during walking increases energy expenditure and alters kinematics (Mendonca et al., 2014; Walden et al., 2023), although the cause of these changes is unclear. These afferents could provide an estimate of energy use localized to a muscle or muscle region, simplifying the credit assignment challenge in comparison to a ‘global’ energy estimate. However, it may also require that the nervous system sum and weight expenditures from various muscles to produce a whole-body estimate of cost, if that metric is ultimately optimized. Group III and IV afferents display different latencies following muscular contraction. Group III afferents are more mechanosensitive, responding within 0.03–0.2 s, although their response is modulated by surrounding metabolites. Group IV afferents are more metabosensitive, displaying slower responses (5–30 s) that may reflect metabolite accumulation (Kaufman and Hayes, 2002; Kaufman et al., 1983, 1984; Mense and Stahnke, 1983). A direct closed-loop test of the ability of these afferents to affect preferred movements has not been pursued (and would be experimentally challenging, given the need to isolate and control the metabolic environment of one or more muscles).

### Proprioceptive mechanosensitive muscle afferents

Mechanoreceptors in the muscles and joints can convey information about the body's posture, movement and exerted forces, which could collectively be used to estimate energy use. For example, muscle spindles that sense length/velocity and Golgi tendon organs that sense force/tension could in combination estimate muscle mechanical work and power, which are reasonable proxies for muscle energy use (Blum et al., 2017; Jami et al., 1985; Windhorst, 2007). These sensors have the advantage that they are both local, providing information at the muscle level, and rapid, with latencies in the order of tens of milliseconds (Cameron et al., 2014). To test proprioceptive influence on energy optimization, Hubbuch et al. (2015) applied vibrations to the Achilles tendons to disrupt muscle spindle accuracy during imperceptible changes to treadmill incline (0 to 2.5%) during walking. Participants showed delays in reaching steady-state gaits compared with controls, suggesting that proprioceptive feedback plays a role in sensing energy cost. However, the small changes in metabolic cost in this paradigm (∼1.2%) and complexity of muscle spindle afferent sensitivity (Blum et al., 2020) leaves unclear the exact role of proprioceptors in sensing energy cost. Future studies could leverage new understandings about lower-limb muscle spindle (Lin et al., 2019; Mildren et al., 2019) and Golgi tendon (Faist et al., 2006; Fallon and Macefield, 2007) response dynamics to further investigate their role in energy optimization.

### Vision for prediction

Vision, accompanied by prior experience, can be used to predict energetically favourable movements. For example, from pictures alone people can identify energy optimal riser heights (Warren, 1984). And, as previously discussed, vision is used to plan navigation strategies that minimize energy cost in advance of walking over complex terrain (Daniels and Burn, 2023; Darici and Kuo, 2022, 2023). To directly test the role of vision in energy minimization, O'Connor and Donelan (2012) used virtual reality to couple and then manipulate the relationship (ratio) between visual flow and walking speed. When exposed to the paradigm, initially participants rapidly adjust their speed to return the visually presented speed back toward their preferred walking speed, but then gradually return to the true energy optimal speed they preferred before the manipulation. Although the authors did not directly measure metabolic expenditure, they conclude that vision is used to rapidly adjust speed toward preferred, and likely complements a slower process that minimizes energy expenditure. How and over what timescale vision-based energetic predictions are updated or recalibrated in response to more permanent changes to the body (for example, motor or sensory injury or decline) is an open question.

### Muscle activation and effort: sensing proxies or distinct objectives?

Muscle activation-based metrics have been proposed as an alternative dominant optimization objective. These metrics, which are often experimentally estimated from surface electromyography of primary and measurable muscles, span a range of computational formulations. In some cases, signals from multiple muscles are weighted based on active muscle volume (estimated from lookup tables of muscle cross-sectional area along with measured electromyography activation), and then summed, providing a more global measure of total activation or effort (Miller et al., 2012; Sheehan and Gottschall, 2013). In other cases, only select muscles are used or cost functions are applied to penalize large activations from any particular muscle, irrespective of size. This provides a more local measure of high activation or fatigue (Ackermann and van den Bogert, 2010; Amann, 2011; Brennan et al., 2019; McDonald et al., 2022). In other words, global effort-based objectives represent total activation associated with the exercise, while local fatigue-based objectives capture high activations from select muscles. Although effort-based muscle activation metrics are sometimes framed as an alternative objective to energy (McDonald et al., 2022), they can be viewed as a promising candidate for sensing energy use. These measures are related to the fraction of metabolically active muscle and can provide local and rapid information (even in advance of a movement if an efference copy is used). Indeed, in most naturally occurring movements, these optima are likely coincident (Brennan et al., 2019; McDonald et al., 2022; Miller et al., 2012). Fatigue-based muscle activation metrics, which are more agnostic to total effort or expenditure, may be better framed as a distinct objective.

Both experiments and computational modelling have been used to investigate whether muscle activation-based metrics or metabolic energy expenditure are better (or distinct) optimization criteria in human movement. In naturally occurring movements, such as steady-state walking, running and cycling, experimentally measured muscle activation metrics and metabolic energy expenditure often change in unison and cannot be dissociated from one another (Brennan et al., 2019; McDonald et al., 2022; Miller et al., 2012). This suggests the nervous system may use muscle activation as a proxy for energy use. In musculoskeletal simulations of human gait, minimizing total muscle activation tends to outperform simulations that minimize energy cost alone (Ackermann and van den Bogert, 2010; Gidley et al., 2019; Miller et al., 2012) (although existing energy models are error prone; Koelewijn et al., 2019) and may underestimate cyclic muscle activation costs (Doke and Kuo, 2007; van der Zee and Kuo, 2021). As previously discussed, McDonald et al. (2022) decoupled these metrics using a forced-choice paradigm and found that participants chose an incline gait, despite it resulting in higher whole-body metabolic cost, over a ‘crouch gait’, potentially to avoid local muscle fatigue. This could be viewed as one piece of evidence that muscle activation-based objectives are optimized during gait, and whole-body metabolic cost is not. Although numerous formulations of other objective functions could explain locomotor behaviours in particular contexts and tasks, there is a fundamental evolutionary rationale for minimizing energy, given that calorie scarcity can threaten survival. Another interpretation is that this decoupling is not often naturally encountered and has, in a sense, tricked the sensory system. Finally, it is also possible that preferred gaits may differ when a person is asked to make an explicit choice. Participants optimizing for energy expenditure appear to do so implicitly, being unaware of their gait adaptation and unable to articulate their new preferences (McAllister et al., 2021). Overall, it remains unclear whether muscle activation is used to estimate whole-body energy expenditure/effort or instead provides a distinct fatigue-based objective.

### Multisensory integration in energy optimization

It is logical that we would use several, or all available, sensory signals to estimate energy in order to optimize our movements – exploiting the advantages and mitigating the disadvantages of each in a given context (Fig. 4). Although all aforementioned sensors could be argued to sense a ‘proxy’ for energy, some are certainly more direct than others, and are therefore less likely to be erroneous in novel contexts. Each sensor's dynamics are also associated with differing latencies and may therefore inform different stages of the optimization process. Some sensors provide information about local, muscle-level energetics, while others provide a more global, whole-body measure. The ability to sense changes in energy use with varying directness, latencies and resolution may help inform the optimization process across a range of contexts and provide redundancy in the face of sensory disruption. Given that all sensory pathways are subject to latencies and some level of noise, the nervous system may perform a form of state estimation, where information from multiple sensory pathways is used alongside an internal model to best predict energy state. Understanding whether or how these sensory cues are weighed across our lifespan or after injury may have important implications in facilitating healthy aging and rehabilitation.

## Applications in assistive device design and adoption

**Fig. 5.**
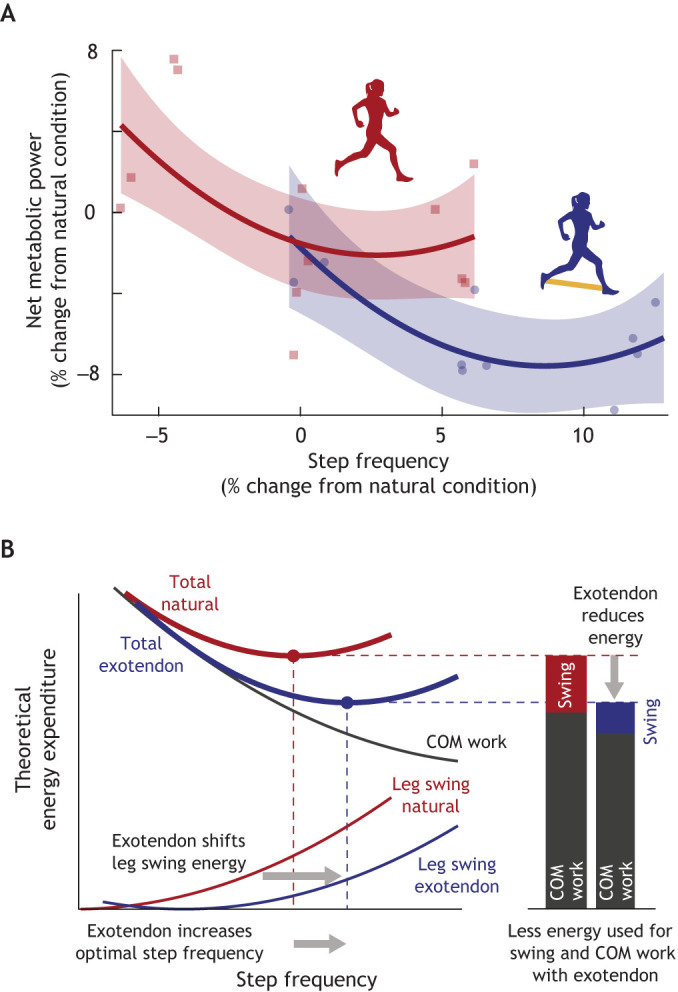
**Relevance of behavioural energetics in assistive device design.** (A) Compared with natural running (red), running with the exotendon (blue) increased the energy optimal step frequency and decreased metabolic energy expenditure*.* Red squares and blue circles represent individual participant data points; lines are quadratic fits for illustrative purposes. Shading shows the 95% confidence intervals of the fits. (B) Theoretical diagram outlining the hypothesized mechanism of energy savings when running with an exotendon, where both swing and stance costs (COM work) are decreased. Reprinted from Simpson et al. (2019). See Supplementary Materials and Methods for additional figure generation details.

Energy-driven preferences may complicate the intended function of devices designed to augment human movement. For assistive exoskeletons and prosthetics, parameters such as actuation timing or device stiffness are often tuned to reduce metabolic cost (Dellon and Matsuoka, 2007; Dollar and Herr, 2008; Sawicki et al., 2020), mimicking a dominant objective of the nervous system (Selinger et al., 2015, 2019). This can be done heuristically through iterative testing, or more recently using human-in-the-loop optimization (Ding et al., 2018; Malcolm et al., 2013; Zhang et al., 2017). In a simplistic view, a well-designed and controlled device applies joint torques or performs work to replace that typically performed by active muscle, reducing metabolic cost. An implicit assumption is often that gross gait kinematics with the device are unchanged; however, when implemented, these devices can create unexpected and complex interactions between the body's dynamics and the user's adaptive strategies. For example, along with Hawkes and colleagues, Selinger tested whether human running economy could be improved by adding a simple spring between the swinging legs of a human runner (Simpson et al., 2019). When wearing the device, costs associated with leg swing decrease (as expected), but runners also increase their step frequency (shorten their stride) to realize larger savings associated with performing less work on the centre of mass during stance (Fig. 5). In fact, depending on the landscape slopes (or marginal costs) of swing and stance gait components, one could design a device to reduce the cost of swing but find these costs increase when the user adapts to minimize total energy expenditure. This principle can be generalized to other gait components and devices, illustrating that human preferences for energy optimal gaits should be carefully considered when modelling, designing and controlling devices to assist locomotion (Koelewijn and Selinger, 2022).

Given that adaptation toward energy optimal gaits can be complex and time-consuming, a deeper understanding of how training and feedback can expedite the process would benefit assistive device designers and users. Although many have focused on the benefits of optimizing exoskeleton device parameters to achieve energy savings, users' internal optimization (evidenced through motor adaptation and learning in response to the device) appears to play a substantial role – accounting for one-quarter to two-thirds of metabolic cost reductions by some estimates (Poggensee and Collins, 2021; Zhang et al., 2017). As previously discussed, a user's internal gait variability and external variation in training conditions may enhance the magnitude and rate of adaptation toward energy optimal gaits (Abram et al., 2022; Poggensee and Collins, 2021). Visual feedback of joint kinematics can also aid users in more quickly adapting toward cost reductions (Kim et al., 2022). A large body of motor control and learning literature exists that can provide insight into how practice and feedback can be optimally designed, leveraging concepts such as motor consolidation, massed and distributed practice, and internal and external feedback (Donovan and Radosevich, 1999; Janacsek and Nemeth, 2012; Wulf, 2013). Applying these concepts could reduce the time and experience required for users to adapt to novel assistive devices and lead to greater cost reductions.

## Applications in rehabilitation and training

**Fig. 6.**
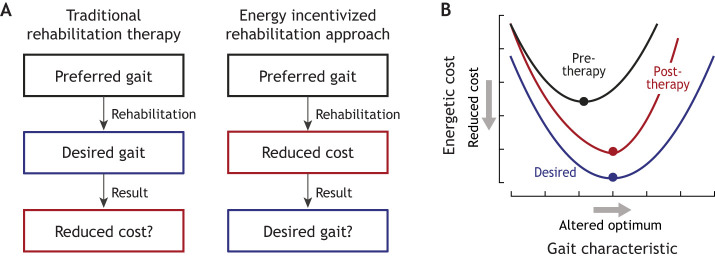
**Relevance of behavioural energetics in rehabilitation and training.** (A) Conceptual diagram illustrating the difference between traditional and energy incentivized rehabilitation approaches. (B) Theoretical outcome of an energy incentivized rehabilitation approach, where the cost landscape is intentionally altered to realign the energy optima with the desired gait characteristic.

An ability to intentionally and precisely alter energy optima, and consequently preferred gaits, has important implications in clinical rehabilitation and athletic training. Although we adapt toward energy optimal behaviours, we have scant evidence that cost landscapes can be more permanently altered with training interventions. With age, injury and obesity, preferred walking speed tends to decrease, often resulting in higher cost of transport (cost per unit distance) (Boyer et al., 2023; Browning and Kram, 2005; Das Gupta et al., 2019; Schrack et al., 2012; Tesio et al., 1991; Zamparo et al., 1995). In addition, for a given gait speed, the metabolic cost of walking tends to be higher (cost per unit time) (Christiansen et al., 2009; Hortobagyi et al., 2011; Imms et al., 1976). This may be either due to neurological or mechanical changes to the body, or because other objectives, such as reducing pain or increasing stability, are now more important (Awad et al., 2023, 2015; Coyle et al., 2019; Gast et al., 2019; Matsubara et al., 2015; Van Hooren et al., 2024). Traditional rehabilitation strategies tend to directly target the desired kinematically ‘normal’ gait, often through repetitive practice under the guidance of a therapist (Fig. 6A). The expected outcome is that the desired gait will eventually be adopted, and high energetic costs will decrease (da Cunha et al., 2002; Reisman et al., 2013). An alternative approach recently tested by Roemmich et al. (2019) directly targets the energetic consequences of movement to incentivize adaptations (Fig. 6B). Here, the hypothesis is that the desired gait will be naturally adopted by the individual, leading to more effective and enduring rehabilitation. Roemmich et al. (2019) were the first to test this novel therapeutic approach using their previously discussed paradigm that linked walking speed to step length symmetry. They found that most post-stroke participants in the study adapted toward a more symmetrical gait when it was made more economical, illustrating that people will alter clinically relevant features of walking to save energy (Roemmich et al., 2019). Although it is unclear whether naturally preferred step asymmetries in this population are predominantly driven by energy minimization (Nguyen et al., 2020), it appears that energetic incentives can still affect gait change. Analogous, overground paradigms using wearable exoskeletons could be designed to target symmetry or other common clinically relevant outcomes, such as walking speed (Lakmazaheri et al., 2024), joint loading and pain (Raitor et al., 2024), or stability and fall risk (Raitor et al., 2024).

Paradigms relying on external mechatronic systems to alter energetic landscapes can help answer an important fundamental question: will a population of interest adapt toward clinically relevant gait features when energetically incentivized to do so? An important related question is: how can exercise training or rehabilitation alter landscapes toward desired gaits over time without these complex external systems? There is evidence of some alignment between preferred and energy optimal gaits across populations of various ages (DeJaeger et al., 2001; Mian et al., 2006), body types (Browning et al., 2006; Browning and Kram, 2005) and mobility challenges (Herr and Grabowski, 2012; Roemmich et al., 2019). However, to our knowledge no study has examined how, or whether, training can affect energy optimal gaits over short timescales and within an individual. A deeper understanding of the relationships between muscle function, energetics and mechanics may allow for targeted exercise interventions that align energetic incentives with desired goals, kinematic or otherwise. Whether this is possible and whether it demands a radically different approach than targeting desired kinematics remains an open question.

## Existing challenges and future opportunities

We identified several exciting opportunities for future work in behavioural energetics. Within each of the preceding sections, knowledge gaps emerged that we think are of greatest importance. (1) There is a need for experiments and methods to systematically quantify the relative importance of energy minimization and other key objectives (see ‘Evidence that preferred behaviours coincide with energy optima’). Here, as some have begun to do (Carlisle and Kuo, 2023; Skinner et al., 2015), energy expenditure can be used as a ‘common currency’, where trade-offs with other objectives can be assessed based on the number of calories one is willing to forgo. An ability to quantify how trade-offs change across tasks, environments and within individuals is of both fundamental and applied importance, particularly in free-living settings. (2) There is a need to better understand the mechanisms that underlie energy optimization (see ‘Evidence that behaviours are adapted toward energy optima’). While there is emerging evidence of the importance of varied experience, either through internal movement variability (Abram et al., 2022; Selinger et al., 2019) or external training (Kim et al., 2022; Poggensee and Collins, 2021), how the nervous system performs this optimization in real-time – navigating an expanse of possible movements to arrive at the optimal solution – is largely unknown. (3) There is a need for experiments to examine what sensory pathways inform energy optimization (see ‘Candidate sensory mechanisms of energy use’). While several candidate pathways have been identified, direct evidence of their role in real-time movement optimization is often lacking. Moreover, while integrating signals from multiple sensory sources would confer many advantages, little is known about whether or how this is done, and how weighting of signals may change with time, task and context. (4) There is a need to account for how energy-driven preferences will complicate assistive device function and take a more systematic and theory grounded approach to user training (see ‘Applications in assistive device design and adoption’). Here, a renewed focus on the human side of this human–machine interaction, including the incorporation of motor learning principles, may be the key to continued performance improvements and cost reductions. (5) There is a need to further explore whether energy-driven preferences can be leveraged to alter movements toward clinically relevant outcomes (see ‘Applications in rehabilitation and training’). Exciting new work shows that altering energy optimal behaviour is possible with mechatronic training systems or assistive devices (Abram et al., 2019; Roemmich et al., 2019; Selinger et al., 2015; Simha et al., 2021; Zhang et al., 2017). It would also be useful to understand whether targeted rehabilitation and training interventions can achieve this in a more permanent fashion and without external devices.

Human behavioural energetics offers a lens through which we can understand a broad range of human locomotor behaviours. While evidence of our preference for energy optimal gaits has existed for decades, new research is revealing its relevance across a surprising array of dynamic tasks and complex environments. Although much remains to be understood, new experimental paradigms are allowing researchers to study this process in real-time and are creating opportunities to investigate the sensory pathways and mechanisms that underlie energy optimization. These fundamental insights could have important applications in designing novel rehabilitation strategies and assistive devices to improve human mobility.

## Supplementary Material

10.1242/jexbio.248125_sup1 Supplementary information

## References

[JEB248125C1] Abe, D., Fukuoka, Y. and Horiuchi, M. (2019). Why do we transition from walking to running? Energy cost and lower leg muscle activity before and after gait transition under body weight support. *PeerJ* 7, e8290. 10.7717/peerj.8290 31871846 PMC6924320

[JEB248125C2] Abram, S. J., Poggensee, K. L., Sanchez, N., Simha, S. N., Finley, J. M., Collins, S. H. and Donelan, J. M. (2022). General variability leads to specific adaptation toward optimal movement policies. *Curr. Biol.* 32, 2222-2232.e5. 10.1016/j.cub.2022.04.015 35537453 PMC9504978

[JEB248125C3] Abram, S. J., Selinger, J. C. and Donelan, J. M. (2019). Energy optimization is a major objective in the real-time control of step width in human walking. *J. Biomech.* 91, 85-91. 10.1016/j.jbiomech.2019.05.010 31151794

[JEB248125C4] Ackermann, M. and van den Bogert, A. J. (2010). Optimality principles for model-based prediction of human gait. *J. Biomech.* 43, 1055-1060. 10.1016/j.jbiomech.2009.12.012 20074736 PMC2849893

[JEB248125C5] Adreani, C. M. and Kaufman, M. P. (1998). Effect of arterial occlusion on responses of group III and IV afferents to dynamic exercise. *J. Appl. Physiol. (1985)* 84, 1827-1833. 10.1152/jappl.1998.84.6.1827 9609773

[JEB248125C6] Alexander, R. M. (1996). *Optima for Animals: Revised Edition*. Princeton, NJ: Princeton University Press.

[JEB248125C7] Alexander, R. M. (2001). Design by numbers. *Nature* 412, 591. 10.1038/35088155 11493899

[JEB248125C8] Amann, M. (2011). *Central and Peripheral Fatigue: Interaction During Cycling Exercise in Humans*, Vol. 43, pp. 2039-2045: Lippincott Williams and Wilkins. 10.1249/MSS.0b013e31821f59ab 21502884

[JEB248125C9] Amann, M., Blain, G. M., Proctor, L. T., Sebranek, J. J., Pegelow, D. F. and Dempsey, J. A. (2010). Group III and IV muscle afferents contribute to ventilatory and cardiovascular response to rhythmic exercise in humans. *J. Appl. Physiol. (1985)* 109, 966-976. 10.1152/japplphysiol.00462.2010 20634355 PMC2963332

[JEB248125C10] Amann, M., Runnels, S., Morgan, D. E., Trinity, J. D., Fjeldstad, A. S., Wray, D. W., Reese, V. R. and Richardson, R. S. (2011). On the contribution of group III and IV muscle afferents to the circulatory response to rhythmic exercise in humans. *J. Physiol. (Lond)* 589, 3855-3866. 10.1113/jphysiol.2011.209353 21646407 PMC3171890

[JEB248125C11] Arellano, C. J. and Kram, R. (2011). The effects of step width and arm swing on energetic cost and lateral balance during running. *J. Biomech.* 44, 1291-1295. 10.1016/j.jbiomech.2011.01.002 21316058

[JEB248125C12] Arellano, C. J. and Kram, R. (2014a). The metabolic cost of human running: is swinging the arms worth it? *J. Exp. Biol.* 217, 2456-2461. 10.1242/jeb.100420 25031455

[JEB248125C13] Arellano, C. J. and Kram, R. (2014b). Partitioning the metabolic cost of human running: a task-by-task approach. *Integr. Comp. Biol.* 54, 1084-1098. 10.1093/icb/icu033 24838747 PMC4296200

[JEB248125C14] Atzler, E. and Herbst, R. (1928). Arbeitsphysiologische Studien III. *Pflugers Arch.* 215, 292.

[JEB248125C15] Awad, L. N., Knarr, B. A., Kudzia, P. and Buchanan, T. S. (2023). The interplay between walking speed, economy, and stability after stroke. *J. Neurol. Phys. Ther.* 47, 75-83. 10.1097/NPT.0000000000000431 36867550 PMC10033356

[JEB248125C16] Awad, L. N., Palmer, J. A., Pohlig, R. T., Binder-Macleod, S. A. and Reisman, D. S. (2015). Walking speed and step length asymmetry modify the energy cost of walking after stroke. *Neurorehabil. Neural Repair* 29, 416-423. 10.1177/1545968314552528 25288581 PMC4385745

[JEB248125C17] Band, D. M., Wolff, C. B., Ward, J., Cochrane, G. M. and Prior, J. (1980). Respiratory oscillations in arterial carbon dioxide tension as a control signal in exercise. *Nature* 283, 84-85. 10.1038/283084a0 7350529

[JEB248125C18] Baroudi, L., Barton, K., Cain, S. M. and Shorter, K. A. (2024). Understanding the influence of context on real-world walking energetics. *J. Exp. Biol.* 227, jeb246181. 10.1242/jeb.246181 38853583

[JEB248125C19] Bellman, R. (1952). On the theory of dynamic programming. *Proc. Natl. Acad. Sci. U.S.A* 38, 716-719. 10.1073/pnas.38.8.716 16589166 PMC1063639

[JEB248125C20] Bernstein, N. (1966). *The Co-ordination and Regulation of Movements*. Oxford, UK: Pergamon Press.

[JEB248125C21] Bertram, J. E. and Ruina, A. (2001). Multiple walking speed-frequency relations are predicted by constrained optimization. *J. Theor. Biol.* 209, 445-453. 10.1006/jtbi.2001.2279 11319893

[JEB248125C22] Black, A. M., McCloskey, D. I. and Torrance, R. W. (1971). The responses of carotid body chemoreceptors in the cat to sudden changes of hypercapnic and hypoxic stimuli. *Respir. Physiol.* 13, 36-49. 10.1016/0034-5687(71)90063-6 5112829

[JEB248125C23] Blum, K. P., Campbell, K. S., Horslen, B. C., Nardelli, P., Housley, S. N., Cope, T. C. and Ting, L. H. (2020). Diverse and complex muscle spindle afferent firing properties emerge from multiscale muscle mechanics. *Elife* 9, e55177. 10.7554/eLife.55177 33370235 PMC7769569

[JEB248125C24] Blum, K. P., Lamotte D'Incamps, B., Zytnicki, D. and Ting, L. H. (2017). Force encoding in muscle spindles during stretch of passive muscle. *PLoS Comput. Biol.* 13, e1005767. 10.1371/journal.pcbi.1005767 28945740 PMC5634630

[JEB248125C25] Bornstein, M. H. and Bornstein, H. G. (1976). The pace of life. *Nature* 259, 557-559. 10.1038/259557a0

[JEB248125C26] Boyer, K. A., Hayes, K. L., Umberger, B. R., Adamczyk, P. G., Bean, J. F., Brach, J. S., Clark, B. C., Clark, D. J., Ferrucci, L., Finley, J. et al. (2023). Age-related changes in gait biomechanics and their impact on the metabolic cost of walking: Report from a National Institute on Aging workshop. *Exp. Gerontol.* 173, 112102. 10.1016/j.exger.2023.112102 36693530 PMC10008437

[JEB248125C27] Bramble, D. M. and Lieberman, D. E. (2004). Endurance running and the evolution of Homo. *Nature* 432, 345-352. 10.1038/nature03052 15549097

[JEB248125C28] Brennan, S. F., Cresswell, A. G., Farris, D. J. and Lichtwark, G. A. (2019). The effect of cadence on the mechanics and energetics of constant power cycling. *Med. Sci. Sports Exerc.* 51, 941-950. 10.1249/MSS.0000000000001863 30531486

[JEB248125C29] Brisswalter, J., Hausswirth, C., Smith, D., Vercruyssen, F. and Vallier, J. M. (2000). Energetically optimal cadence vs. freely-chosen cadence during cycling: effect of exercise duration. *Int. J. Sports Med.* 21, 60-64. 10.1055/s-2000-8857 10683101

[JEB248125C30] Brockway, J. M. (1987). Derivation of formulae used to calculate energy expenditure in man. *Hum. Nutr. Clin. Nutr.* 41, 463-471. 3429265

[JEB248125C31] Brooks, G., Fahey, T. and White, T. (2005). Basics of metabolism. In *Exercise Physiology: Human Bioenergetics and Its Application*, pp. 43-58. London: McGraw-Hill Education.

[JEB248125C32] Brown, G. L., Seethapathi, N. and Srinivasan, M. (2021). A unified energy-optimality criterion predicts human navigation paths and speeds. *Proc. Natl. Acad. Sci. U.S.A* 118, e2020327118. 10.1073/pnas.2020327118 34266945 PMC8307777

[JEB248125C33] Browning, R. C., Baker, E. A., Herron, J. A. and Kram, R. (2006). Effects of obesity and sex on the energetic cost and preferred speed of walking. *J. Appl. Physiol. (1985)* 100, 390-398. 10.1152/japplphysiol.00767.2005 16210434

[JEB248125C34] Browning, R. C. and Kram, R. (2005). Energetic cost and preferred speed of walking in obese vs. normal weight women. *Obes. Res.* 13, 891-899. 10.1038/oby.2005.103 15919843

[JEB248125C35] Cameron, B. D., de la Malla, C. and Lopez-Moliner, J. (2014). The role of differential delays in integrating transient visual and proprioceptive information. *Front. Psychol.* 5, 50. 24550870 10.3389/fpsyg.2014.00050 PMC3910305

[JEB248125C36] Carlisle, R. E. and Kuo, A. D. (2023). Optimization of energy and time predicts dynamic speeds for human walking. *eLife* 12, e81939. 10.7554/eLife.81939 36779697 PMC10030114

[JEB248125C37] Carrier, D. R., Kapoor, A. K., Kimura, T., Nickels, M. K., Scott, E. C., So, J. K. and Trinkaus, E. (1984). The energetic paradox of human running and hominid evolution [and Comments and Reply]. *Curr. Anthropol* 25, 483-495. 10.1086/203165

[JEB248125C38] Casaburi, R., Whipp, B. J., Wasserman, K., Beaver, W. L. and Koyal, S. N. (1977). Ventilatory and gas exchange dynamics in response to sinusoidal work. *J. Appl. Physiol. Respir. Environ. Exerc. Physiol.* 42, 300-311. 838654 10.1152/jappl.1977.42.2.300

[JEB248125C39] Cher, P. H., Stewart, I. B. and Worringham, C. J. (2015). Minimum cost of transport in human running is not ubiquitous. *Med. Sci. Sports Exerc* 47, 307-314. 10.1249/MSS.0000000000000421 24977694

[JEB248125C40] Choi, J. T., Vining, E. P., Reisman, D. S. and Bastian, A. J. (2009). Walking flexibility after hemispherectomy: split-belt treadmill adaptation and feedback control. *Brain* 132, 722-733. 10.1093/brain/awn333 19074191 PMC2664447

[JEB248125C41] Christiansen, C. L., Schenkman, M. L., McFann, K., Wolfe, P. and Kohrt, W. M. (2009). Walking economy in people with Parkinson's disease. *Mov. Disord* 24, 1481-1487. 10.1002/mds.22621 19441128 PMC2744295

[JEB248125C42] Courter, R. J., Alvarez, E., Enoka, R. M. and Ahmed, A. A. (2023). Metabolic costs of walking and arm reaching in persons with mild multiple sclerosis. *J. Neurophysiol.* 129, 819-832. 10.1152/jn.00373.2022 36883754 PMC10085565

[JEB248125C43] Coyle, P. C., Pugliese, J. M., Sions, J. M., Eskander, M. S., Schrack, J. A. and Hicks, G. E. (2019). Pain provocation and the energy cost of walking: a matched comparison study of older adults with and without chronic low back pain with radiculopathy. *J. Geriatr. Phys. Ther.* 42, E97-E104. 10.1519/JPT.0000000000000212 30998562 PMC6783346

[JEB248125C44] Crisafulli, A., Piras, F., Filippi, M., Piredda, C., Chiappori, P., Melis, F., Milia, R., Tocco, F. and Concu, A. (2011). Role of heart rate and stroke volume during muscle metaboreflex-induced cardiac output increase: differences between activation during and after exercise. *J. Physiol. Sci.* 61, 385-394. 10.1007/s12576-011-0163-x 21796398 PMC10717214

[JEB248125C45] da Cunha, I. T., Jr, Lim, P. A., Qureshy, H., Henson, H., Monga, T. and Protas, E. J. (2002). Gait outcomes after acute stroke rehabilitation with supported treadmill ambulation training: a randomized controlled pilot study. *Arch. Phys. Med. Rehabil.* 83, 1258-1265. 10.1053/apmr.2002.34267 12235606

[JEB248125C46] Dahan, A., DeGoede, J., Berkenbosch, A. and Olievier, I. C. (1990). The influence of oxygen on the ventilatory response to carbon dioxide in man. *J. Physiol. (Lond.)* 428, 485-499. 10.1113/jphysiol.1990.sp018223 2121961 PMC1181658

[JEB248125C47] Daniels, K. A. J. and Burn, J. F. (2023). Human locomotion over obstacles reveals real-time prediction of energy expenditure for optimized decision-making. *Proc. R. Soc. Lond. B Biol. Sci.* 290, 20230200. 10.1098/rspb.2023.0200 PMC10265010 37312546

[JEB248125C48] Darici, O. and Kuo, A. D. (2022). Humans optimally anticipate and compensate for an uneven step during walking. *eLife* 11, e65402. 10.7554/eLife.65402 35014609 PMC8920505

[JEB248125C49] Darici, O. and Kuo, A. D. (2023). Humans plan for the near future to walk economically on uneven terrain. *Proc. Natl. Acad. Sci. U.S.A* 120, e2211405120. 10.1073/pnas.2211405120 37126717 PMC10175744

[JEB248125C50] Das Gupta, S., Bobbert, M. F. and Kistemaker, D. A. (2019). The metabolic cost of walking in healthy young and older adults - a systematic review and meta analysis. *Sci. Rep.* 9, 9956. 10.1038/s41598-019-45602-4 31292471 PMC6620279

[JEB248125C51] de Rugy, A., Loeb, G. E. and Carroll, T. J. (2012). Muscle coordination is habitual rather than optimal. *J. Neurosci.* 32, 7384-7391. 10.1523/JNEUROSCI.5792-11.2012 22623684 PMC6622296

[JEB248125C52] Dean, J. C. (2013). Proprioceptive feedback and preferred patterns of human movement. *Exerc. Sport Sci. Rev.* 41, 36-43. 10.1097/JES.0b013e3182724bb0 23038242 PMC5997460

[JEB248125C53] DeJaeger, D., Willems, P. A. and Heglund, N. C. (2001). The energy cost of walking in children. *Pflugers Arch.* 441, 538-543. 10.1007/s004240000443 11212218

[JEB248125C54] Dellon, B. and Matsuoka, Y. (2007). Prosthetics, exoskeletons, and rehabilitation [Grand Challenges of Robotics]. *IEEE Robot Autom. Mag.* 14, 30-34. 10.1109/MRA.2007.339622

[JEB248125C55] Diedrich, F. J. and Warren, W. H. Jr. (1995). Why change gaits? Dynamics of the walk-run transition. *J. Exp. Psychol. Hum. Percept. Perform*. 21, 183-202. 10.1037/0096-1523.21.1.183 7707029

[JEB248125C56] Ding, Y., Kim, M., Kuindersma, S. and Walsh, C. J. (2018). Human-in-the-loop optimization of hip assistance with a soft exosuit during walking. *Sci. Robot* 3, eaar5438. 33141683 10.1126/scirobotics.aar5438

[JEB248125C57] Doke, J. and Kuo, A. D. (2007). Energetic cost of producing cyclic muscle force, rather than work, to swing the human leg. *J. Exp. Biol.* 210, 2390-2398. 10.1242/jeb.02782 17575044

[JEB248125C58] Dollar, A. M. and Herr, H. (2008). lower extremity exoskeletons and active orthoses: challenges and state-of-the-art. *IEEE Trans. Robot* 24, 144-158. 10.1109/TRO.2008.915453

[JEB248125C59] Donelan, J. M., Kram, R. and Kuo, A. D. (2001). Mechanical and metabolic determinants of the preferred step width in human walking. *Proc. R. Soc. Lond. B Biol. Sci.* 268, 1985-1992. 10.1098/rspb.2001.1761 PMC1088839 11571044

[JEB248125C60] Donelan, J. M., Kram, R. and Kuo, A. D. (2002). Mechanical work for step-to-step transitions is a major determinant of the metabolic cost of human walking. *J. Exp. Biol.* 205, 3717-3727. 10.1242/jeb.205.23.3717 12409498

[JEB248125C61] Donelan, J. M., Shipman, D. W., Kram, R. and Kuo, A. D. (2004). Mechanical and metabolic requirements for active lateral stabilization in human walking. *J. Biomech.* 37, 827-835. 10.1016/j.jbiomech.2003.06.002 15111070

[JEB248125C62] Donovan, J. J. and Radosevich, D. J. (1999). A meta-analytic review of the distribution of practice effect: Now you see it, now you don't. *J. Appl. Psychol.* 84, 795-805. 10.1037/0021-9010.84.5.795

[JEB248125C63] Elftman, H. (1966). Biomechanics of muscle with particular application to studies of gait. *J. Bone. Joint. Surg. Am.* 48, 363-377. 10.2106/00004623-196648020-00017 5932924

[JEB248125C64] Faist, M., Hoefer, C., Hodapp, M., Dietz, V., Berger, W. and Duysens, J. (2006). In humans Ib facilitation depends on locomotion while suppression of Ib inhibition requires loading. *Brain Res.* 1076, 87-92. 10.1016/j.brainres.2005.12.069 16472783

[JEB248125C65] Fallon, J. B. and Macefield, V. G. (2007). Vibration sensitivity of human muscle spindles and golgi tendon organs. *Muscle Nerve* 36, 21-29. 10.1002/mus.20796 17471568

[JEB248125C66] Ferris, D. P., Sawicki, G. S. and Daley, M. A. (2007). A physiologist's perspective on robotic exoskeletons for human locomotion. *Int. J. Humanoid Robot* 4, 507-528. 10.1142/S0219843607001138 PMC2185037 18185840

[JEB248125C67] Finley, J. M., Bastian, A. J. and Gottschall, J. S. (2013). Learning to be economical: the energy cost of walking tracks motor adaptation. *J. Physiol. (Lond)* 591, 1081-1095. 10.1113/jphysiol.2012.245506 23247109 PMC3591716

[JEB248125C68] Fitzgerald, R. S., Leitner, L. M. and Liaubet, M. J. (1969). Carotid chemoreceptor response to intermittent or sustained stimulation in the cat. *Respir. Physiol.* 6, 395-402. 10.1016/0034-5687(69)90037-1 5778484

[JEB248125C69] Formenti, F., Dockerill, C., Kankanange, L., Zhang, L., Takaishi, T. and Ishida, K. (2019). The effect of pedaling cadence on skeletal muscle oxygenation during cycling at moderate exercise intensity. *Int. J. Sports Med.* 40, 305-311. 10.1055/a-0835-6286 30736073

[JEB248125C70] Forster, H. V., Dunning, M. B., Lowry, T. F., Erickson, B. K., Forster, M. A., Pan, L. G., Brice, A. G. and Effros, R. M. (1993). Effect of asthma and ventilatory loading on arterial PCO2 of humans during submaximal exercise. *J. Appl. Physiol. (1985)* 75, 1385-1394. 10.1152/jappl.1993.75.3.1385 8226555

[JEB248125C71] Gandevia, S. C. (2001). Spinal and supraspinal factors in human muscle fatigue. *Physiol. Rev.* 81, 1725-1789. 10.1152/physrev.2001.81.4.1725 11581501

[JEB248125C72] Gast, K., Kram, R. and Riemer, R. (2019). Preferred walking speed on rough terrain: is it all about energetics? *J. Exp. Biol.* 222, jeb185447. 10.1242/jeb.185447 30910832

[JEB248125C73] Gidley, A. D. and Lankford, D. E. (2021). Cost of transport at preferred walking speeds are minimized while walking on moderately steep incline surfaces. *Hum. Mov. Sci.* 79, 102849. 10.1016/j.humov.2021.102849 34385052

[JEB248125C74] Gidley, A. D., Marsh, A. P. and Umberger, B. R. (2019). Performance criteria for generating predictive optimal control simulations of bicycle pedaling. *Comput. Methods Biomech. Biomed. Eng.* 22, 11-20. 10.1080/10255842.2018.1522535 30398070

[JEB248125C75] Glass, S., Dwyer, G. B. and Medicine, A. C. o. S. (2007). *ACSM's Metabolic Calculations Handbook*. Lippincott Williams & Wilkins.

[JEB248125C76] Gottschall, J. S. and Kram, R. (2003). Energy cost and muscular activity required for propulsion during walking. *J. Appl. Physiol. (1985)* 94, 1766-1772. 10.1152/japplphysiol.00670.2002 12506042

[JEB248125C77] Gottschall, J. S. and Kram, R. (2005). Energy cost and muscular activity required for leg swing during walking. *J. Appl. Physiol. (1985)* 99, 23-30. 10.1152/japplphysiol.01190.2004 16036902

[JEB248125C78] Grabowski, A., Farley, C. T. and Kram, R. (2005). Independent metabolic costs of supporting body weight and accelerating body mass during walking. *J. Appl. Physiol. (1985)* 98, 579-583. 10.1152/japplphysiol.00734.2004 15649878

[JEB248125C79] Gutmann, A. K., Jacobi, B., Butcher, M. T. and Bertram, J. E. A. (2006). Constrained optimization in human running. *J. Exp. Biol.* 209, 622-632. 10.1242/jeb.02010 16449557

[JEB248125C80] Herr, H. M. and Grabowski, A. M. (2012). Bionic ankle-foot prosthesis normalizes walking gait for persons with leg amputation. *Proc. R. Soc. Lond. B Biol. Sci.* 279, 457-464. 10.1098/rspb.2011.1194 PMC3234569 21752817

[JEB248125C81] Heymans, J. F. H. (1927). Sur les modifications directes et sur la régulation réflexe de l'activité du centre respiratoire de la tête isolée du chien. *Arch. Int. Pharmacodyn. Ther.* 33, 273-372.

[JEB248125C82] Holt, K. G., Hamill, J. and Andres, R. O. (1991). Predicting the minimal energy costs of human walking. *Med. Sci. Sports Exerc* 23, 491-498. 1905381

[JEB248125C83] Hortobagyi, T., Finch, A., Solnik, S., Rider, P. and DeVita, P. (2011). Association between muscle activation and metabolic cost of walking in young and old adults. *J. Gerontol. A. Biol. Sci. Med. Sci.* 66, 541-547. 10.1093/gerona/glr008 21345892 PMC3074960

[JEB248125C84] Hoyt, D. F. and Taylor, C. R. (1981). Gait and the energetics of locomotion in horses. *Nature* 292, 239-240. 10.1038/292239a0

[JEB248125C85] Hreljac, A. (1993). Preferred and energetically optimal gait transition speeds in human locomotion. *Med. Sci. Sports Exerc* 25, 1158-1162. 10.1249/00005768-199310000-00012 8231761

[JEB248125C86] Huang, H. J., Kram, R. and Ahmed, A. A. (2012). Reduction of metabolic cost during motor learning of arm reaching dynamics. *J. Neurosci.* 32, 2182-2190. 10.1523/JNEUROSCI.4003-11.2012 22323730 PMC3865509

[JEB248125C87] Hubbuch, J. E., Bennett, B. W. and Dean, J. C. (2015). Proprioceptive feedback contributes to the adaptation toward an economical gait pattern. *J. Biomech.* 48, 2925-2931. 10.1016/j.jbiomech.2015.04.024 25935689 PMC7167590

[JEB248125C88] Hunter, L. C., Hendrix, E. C. and Dean, J. C. (2010). The cost of walking downhill: is the preferred gait energetically optimal? *J. Biomech.* 43, 1910-1915. 10.1016/j.jbiomech.2010.03.030 20399434

[JEB248125C89] Imms, F. J., MacDonald, I. C. and Prestidge, S. P. (1976). Energy expenditure during walking in patients recovering from fractures of the leg. *Scand. J. Rehabil. Med.* 8, 1-9. 935837

[JEB248125C90] Ingraham, K. A., Ferris, D. P. and Remy, C. D. (2019). Evaluating physiological signal salience for estimating metabolic energy cost from wearable sensors. *J. Appl. Physiol. (1985)* 126, 717-729. 10.1152/japplphysiol.00714.2018 30629472 PMC6459384

[JEB248125C91] Ivanenko, Y. P., Dominici, N. and Lacquaniti, F. (2007). *Development of independent walking in toddlers*. *Exerc. Sport Sci. Rev.* 35, 67-73. 10.1249/JES.0b013e31803eafa8 17417053

[JEB248125C92] Jami, L., Petit, J., Proske, U. and Zytnicki, D. (1985). Responses of tendon organs to unfused contractions of single motor units. *J. Neurophysiol.* 53, 32-42. 10.1152/jn.1985.53.1.32 3973662

[JEB248125C93] Janacsek, K. and Nemeth, D. (2012). Predicting the future: from implicit learning to consolidation. *Int. J. Psychophysiol* 83, 213-221. 10.1016/j.ijpsycho.2011.11.012 22154521

[JEB248125C94] Kaufman, M. P. and Hayes, S. G. (2002). The exercise pressor reflex. *Clin. Auton. Res.* 12, 429-439. 10.1007/s10286-002-0059-1 12598947

[JEB248125C95] Kaufman, M. P., Longhurst, J. C., Rybicki, K. J., Wallach, J. H. and Mitchell, J. H. (1983). Effects of static muscular contraction on impulse activity of groups III and IV afferents in cats. *J. Appl. Physiol. Respir. Environ. Exerc. Physiol.* 55, 105-112. 6309712 10.1152/jappl.1983.55.1.105

[JEB248125C96] Kaufman, M. P., Rybicki, K. J., Waldrop, T. G. and Ordway, G. A. (1984). Effect of ischemia on responses of group III and IV afferents to contraction. *J. Appl. Physiol. Respir. Environ. Exerc. Physiol.* 57, 644-650. 6092310 10.1152/jappl.1984.57.3.644

[JEB248125C97] Kim, M., Jeong, H., Kantharaju, P., Yoo, D., Jacobson, M., Shin, D., Han, C. and Patton, J. L. (2022). Visual guidance can help with the use of a robotic exoskeleton during human walking. *Sci. Rep.* 12, 3881. 10.1038/s41598-022-07736-w 35273244 PMC8913727

[JEB248125C98] Kipp, S., Grabowski, A. M. and Kram, R. (2018). What determines the metabolic cost of human running across a wide range of velocities? *J. Exp. Biol.* 221, jeb184218. 10.1242/jeb.184218 30065039

[JEB248125C99] Kistemaker, D. A., Wong, J. D. and Gribble, P. L. (2010). The central nervous system does not minimize energy cost in arm movements. *J. Neurophysiol.* 104, 2985-2994. 10.1152/jn.00483.2010 20884757

[JEB248125C100] Koelewijn, A. D., Heinrich, D. and van den Bogert, A. J. (2019). Metabolic cost calculations of gait using musculoskeletal energy models, a comparison study. *PLoS One* 14, e0222037. 10.1371/journal.pone.0222037 31532796 PMC6750598

[JEB248125C101] Koelewijn, A. D. and Selinger, J. C. (2022). Predictive simulations to replicate human gait adaptations and energetics with exoskeletons. *IEEE Trans. Neural Syst. Rehabil. Eng.* 30, 1931-1940. 10.1109/TNSRE.2022.3189038 35797329

[JEB248125C102] Kram, R. and Taylor, C. R. (1990). Energetics of running: a new perspective. *Nature* 346, 265-267. 10.1038/346265a0 2374590

[JEB248125C103] Kung, S. M., Fink, P. W., Legg, S. J., Ali, A. and Shultz, S. P. (2018). What factors determine the preferred gait transition speed in humans? A review of the triggering mechanisms. *Hum. Mov. Sci.* 57, 1-12. 10.1016/j.humov.2017.10.023 29121506

[JEB248125C104] Kuo, A. D. (2001). A simple model of bipedal walking predicts the preferred speed-step length relationship. *J. Biomech. Eng.* 123, 264-269. 10.1115/1.1372322 11476370

[JEB248125C105] Kuo, A. D. (2007). The six determinants of gait and the inverted pendulum analogy: A dynamic walking perspective. *Hum. Mov. Sci.* 26, 617-656. 10.1016/j.humov.2007.04.003 17617481

[JEB248125C106] Kuo, A. D. and Donelan, J. M. (2010). Dynamic principles of gait and their clinical implications. *Phys. Ther.* 90, 157-174. 10.2522/ptj.20090125 20023002 PMC2816028

[JEB248125C107] Labaune, O., Deroche, T., Teulier, C. and Berret, B. (2020). Vigor of reaching, walking, and gazing movements: on the consistency of interindividual differences. *J. Neurophysiol.* 123, 234-242. 10.1152/jn.00344.2019 31774359

[JEB248125C108] Lakmazaheri, A., Song, S., Vuong, B. B., Biskner, B., Kado, D. M. and Collins, S. H. (2024). Optimizing exoskeleton assistance to improve walking speed and energy economy for older adults. *J. Neuroeng. Rehabil* 21, 1. 10.1186/s12984-023-01287-5 38167151 PMC10763092

[JEB248125C109] Levine, R. V. and Bartlett, K. (2016). Pace of life, punctuality, and coronary heart disease in six countries. *J. Cross-Cult. Psychol.* 15, 233-255. 10.1177/0022002184015002009

[JEB248125C110] Li, L., Nagy, M., Graving, J. M., Bak-Coleman, J., Xie, G. and Couzin, I. D. (2020). Vortex phase matching as a strategy for schooling in robots and in fish. *Nat. Commun.* 11, 5408. 10.1038/s41467-020-19086-0 33106484 PMC7588453

[JEB248125C111] Lin, D. C., McGowan, C. P., Blum, K. P. and Ting, L. H. (2019). Yank: the time derivative of force is an important biomechanical variable in sensorimotor systems. *J. Exp. Biol.* 222, jeb180414. 10.1242/jeb.180414 31515280 PMC6765171

[JEB248125C112] Loeb, G. E. (2012). Optimal isn't good enough. *Biol. Cybern* 106, 757-765. 10.1007/s00422-012-0514-6 22895830

[JEB248125C113] Long, L. L. and Srinivasan, M. (2013). Walking, running, and resting under time, distance, and average speed constraints: optimality of walk–run–rest mixtures. *J. R. Soc. Interface* 10, 20120980. 10.1098/rsif.2012.0980 23365192 PMC3627106

[JEB248125C114] Lugliani, R., Whipp, B. J., Seard, C. and Wasserman, K. (1971). Effect of Bilateral Carotid-Body Resection on Ventilatory Control at Rest and during Exercise in Man. *N. Engl. J. Med.* 285, 1105-1111. 10.1056/NEJM197111112852002 5095735

[JEB248125C115] Lyons, J., Hansen, S., Hurding, S. and Elliott, D. (2006). Optimizing rapid aiming behaviour: Movement kinematics depend on the cost of corrective modifications. *Exp. Brain Res.* 174, 95-100. 10.1007/s00221-006-0426-6 16575577

[JEB248125C116] Malcolm, P., Derave, W., Galle, S. and De Clercq, D. (2013). A simple exoskeleton that assists plantarflexion can reduce the metabolic cost of human walking. *PLoS One* 8, e56137. 10.1371/journal.pone.0056137 23418524 PMC3571952

[JEB248125C117] Margaria, R., Cerretelli, P., Aghemo, P. and Sassi, G. (1963). Energy cost of running. *J. Appl. Physiol. Respir. Environ. Exerc. Physiol.* 18, 367-370. 10.1152/jappl.1963.18.2.367 13932993

[JEB248125C118] Marsh, A. P. and Martin, P. E. (1993). The association between cycling experience and preferred and most economical cadences. *Med. Sci. Sports Exerc* 25, 1269-1274. 10.1249/00005768-199311000-00011 8289615

[JEB248125C119] Marsh, A. P., Martin, P. E. and Sanderson, D. J. (2000). Is a joint moment-based cost function associated with preferred cycling cadence? *J. Biomech.* 33, 173-180. 10.1016/S0021-9290(99)00155-4 10653030

[JEB248125C120] Matsubara, J. H., Wu, M. and Gordon, K. E. (2015). Metabolic cost of lateral stabilization during walking in people with incomplete spinal cord injury. *Gait Posture* 41, 646-651. 10.1016/j.gaitpost.2015.01.015 25670651 PMC4318628

[JEB248125C121] McAllister, M. J., Blair, R. L., Donelan, J. M. and Selinger, J. C. (2021). Energy optimization during walking involves implicit processing. *J. Exp. Biol.* 224, jeb242655. 10.1242/jeb.242655 34521117

[JEB248125C122] McDonald, K. A., Cusumano, J. P., Hieronymi, A. and Rubenson, J. (2022). Humans trade off whole-body energy cost to avoid overburdening muscles while walking. *Proc. R. Soc. Lond. B Biol. Sci.* 289, 20221189. 10.1098/rspb.2022.1189 PMC9597406 36285498

[JEB248125C123] McDonald, K. A., Cusumano, J. P., Peeling, P. and Rubenson, J. (2019). Multi-objective control in human walking: insight gained through simultaneous degradation of energetic and motor regulation systems. *J. R. Soc. Interface* 16, 20190227. 10.1098/rsif.2019.0227 31506049 PMC6769305

[JEB248125C124] Mendonca, G. V., Vaz, J. R., Teixeira, M. S., Gracio, T. and Pezarat-Correia, P. (2014). Metabolic cost of locomotion during treadmill walking with blood flow restriction. *Clin. Physiol. Funct. Imaging* 34, 308-316. 10.1111/cpf.12098 24237757

[JEB248125C125] Menier, D. R. and Pugh, L. G. (1968). The relation of oxygen intake and velocity of walking and running, in competition walkers. *J. Physiol. (Lond.)* 197, 717-721. 10.1113/jphysiol.1968.sp008584 5666183 PMC1351758

[JEB248125C126] Mense, S. and Stahnke, M. (1983). Responses in muscle afferent fibres of slow conduction velocity to contractions and ischaemia in the cat. *J. Physiol. (Lond.)* 342, 383-397. 10.1113/jphysiol.1983.sp014857 6631740 PMC1193965

[JEB248125C127] Mercier, J., Le Gallais, D., Durand, M., Goudal, C., Micallef, J. P. and Prefaut, C. (1994). Energy expenditure and cardiorespiratory responses at the transition between walking and running. *Eur. J. Appl. Physiol.* 69, 525-529. 10.1007/BF00239870 7713073

[JEB248125C128] Mian, O. S., Thom, J. M., Ardigo, L. P., Narici, M. V. and Minetti, A. E. (2006). Metabolic cost, mechanical work, and efficiency during walking in young and older men. *Acta Physiol.* 186, 127-139. 10.1111/j.1748-1716.2006.01522.x 16497190

[JEB248125C129] Mildren, R. L., Peters, R. M., Carpenter, M. G., Blouin, J. S. and Inglis, J. T. (2019). Soleus single motor units show stronger coherence with Achilles tendon vibration across a broad bandwidth relative to medial gastrocnemius units while standing. *J. Neurophysiol.* 122, 2119-2129. 10.1152/jn.00352.2019 31553669 PMC6879960

[JEB248125C130] Miller, M. J. and Tenney, S. M. (1975). Hyperoxic hyperventilation in carotid-deafferented cats. *Respir. Physiol.* 23, 23-30. 10.1016/0034-5687(75)90068-7 1129548

[JEB248125C131] Miller, R. H., Umberger, B. R., Hamill, J. and Caldwell, G. E. (2012). Evaluation of the minimum energy hypothesis and other potential optimality criteria for human running. *Proc. R. Soc. Lond. B Biol. Sci.* 279, 1498-1505. 10.1098/rspb.2011.2015 PMC3282349 22072601

[JEB248125C132] Minetti, A. E., Ardigo, L. P. and Saibene, F. (1994). The transition between walking and running in humans: metabolic and mechanical aspects at different gradients. *Acta Physiol. Scand.* 150, 315-323. 10.1111/j.1748-1716.1994.tb09692.x 8010138

[JEB248125C133] Minetti, A. E., Capelli, C., Zamparo, P., Di Prampero, P. E. and Saibene, F. (1995). Effects of stride frequency on mechanical power and energy expenditure of walking. *Med. Sci. Sports Exerc* 27, 1194-1202. 10.1249/00005768-199508000-00014 7476065

[JEB248125C134] Minsky, M. (1961). Steps toward Artificial Intelligence. *Proc. IRE* 49, 8-30. 10.1109/JRPROC.1961.287775

[JEB248125C135] Mohammad, M., Hijleh, A. A., Phillips, G., McAllister, M. J. and Selinger, J. C. (2023). Estimating energy expenditure using wearable sensors during indoor and outdoor locomotion. *International Society on Biomechanics*, pp. 485. Fukuoka, Japan.

[JEB248125C136] Molen, N. M., Rozendale, R. H. and Boon, W. (1972). Graphic representation of the relationship between oxygen consumption and characteristics of normal gait of the human male. *Proc. K Ned Akad Wet C* 75, 305-314. 4263757

[JEB248125C137] Morton, S. M. and Bastian, A. J. (2006). Cerebellar contributions to locomotor adaptations during splitbelt treadmill walking. *J. Neurosci.* 26, 9107-9116. 10.1523/JNEUROSCI.2622-06.2006 16957067 PMC6674518

[JEB248125C138] Nguyen, T. M., Jackson, R. W., Aucie, Y., de Kam, D., Collins, S. H. and Torres-Oviedo, G. (2020). Self-selected step length asymmetry is not explained by energy cost minimization in individuals with chronic stroke. *J. Neuroeng. Rehabil* 17, 119. 10.1186/s12984-020-00733-y 32847596 PMC7450572

[JEB248125C139] O'Connor, S. M. and Donelan, J. M. (2012). Fast visual prediction and slow optimization of preferred walking speed. *J. Neurophysiol.* 107, 2549-2559. 10.1152/jn.00866.2011 22298829

[JEB248125C140] O'Connor, S. M., Wong, J. D. and Donelan, J. M. (2016). A generalized method for controlling end-tidal respiratory gases during nonsteady physiological conditions. *J. Appl. Physiol. (1985)* 121, 1363-1378. 10.1152/japplphysiol.00818.2016 27633735

[JEB248125C141] Orendurff, M. S., Schoen, J. A., Bernatz, G. C., Segal, A. D. and Klute, G. K. (2008). How humans walk: bout duration, steps per bout, and rest duration. *J. Rehabil. Res. Dev.* 45, 1077-1089. 10.1682/JRRD.2007.11.0197 19165696

[JEB248125C142] Peyre-Tartaruga, L. A., Dewolf, A. H., di Prampero, P. E., Fabrica, G., Malatesta, D., Minetti, A. E., Monte, A., Pavei, G., Silva-Pereyra, V., Willems, P. A. et al. (2021). Mechanical work as a (key) determinant of energy cost in human locomotion: recent findings and future directions. *Exp. Physiol.* 106, 1897-1908. 10.1113/EP089313 34197674

[JEB248125C143] Peyrot, N., Thivel, D., Isacco, L., Morin, J. B., Belli, A. and Duche, P. (2012). Why does walking economy improve after weight loss in obese adolescents? *Med. Sci. Sports Exerc* 44, 659-665. 10.1249/MSS.0b013e318236edd8 21986806

[JEB248125C144] Poggensee, K. L. and Collins, S. H. (2021). How adaptation, training, and customization contribute to benefits from exoskeleton assistance. *Sci. Robot.* 6, eabf1078. 10.1126/scirobotics.abf1078 34586837

[JEB248125C145] Poole, D. C. and Richardson, R. S. (1997). Determinants of Oxygen Uptake. *Sports Med.* 24, 308-320. 10.2165/00007256-199724050-00003 9368277

[JEB248125C146] Price, M., Huber, M. E. and Hoogkamer, W. (2023). Minimum effort simulations of split-belt treadmill walking exploit asymmetry to reduce metabolic energy expenditure. *J. Neurophysiol.* 129, 900-913. 10.1152/jn.00343.2022 36883759 PMC10110733

[JEB248125C147] Raitor, M., Ruggles, S. W., Delp, S. L., Liu, C. K. and Collins, S. H. (2024). Lower-limb exoskeletons appeal to both clinicians and older adults, especially for fall prevention and joint pain reduction. *IEEE Trans. Neural Syst. Rehabil. Eng.* 32, 1577-1585. 10.1109/TNSRE.2024.3381979 38536680

[JEB248125C148] Ralston, H. J. (1958). Energy-speed relation and optimal speed during level walking. *Int. Z. Angew. Physiol.* 17, 277-283. 13610523 10.1007/BF00698754

[JEB248125C149] Rathkey, J. K. and Wall-Scheffler, C. M. (2017). People choose to run at their optimal speed. *Am. J. Phys. Anthropol.* 163, 85-93. 10.1002/ajpa.23187 28195301

[JEB248125C150] Raynor, A. J., Yi, C. J., Abernethy, B. and Jong, Q. J. (2002). Are transitions in human gait determined by mechanical, kinetic or energetic factors? *Hum. Mov. Sci.* 21, 785-805. 10.1016/S0167-9457(02)00180-X 12620720

[JEB248125C151] Reisman, D. S., Binder-MacLeod, S. and Farquhar, W. B. (2013). Changes in metabolic cost of transport following locomotor training poststroke. *Top. Stroke Rehabil* 20, 161-170. 10.1310/tsr2002-161 23611857 PMC4104066

[JEB248125C152] Reisman, D. S., Block, H. J. and Bastian, A. J. (2005). Interlimb coordination during locomotion: what can be adapted and stored? *J. Neurophysiol.* 94, 2403-2415. 10.1152/jn.00089.2005 15958603

[JEB248125C153] Riveros-Matthey, C. D., Carroll, T. J., Connick, M. J. and Lichtwark, G. A. (2023). An in-silico investigation of the effect of changing cycling crank power and cadence on muscle energetics and active muscle volume. *bioRxiv* 2023.09.18.558368. 10.1016/j.jbiomech.2025.112530 39837154

[JEB248125C154] Rock, C. G., Marmelat, V., Yentes, J. M., Siu, K. C. and Takahashi, K. Z. (2018). Interaction between step-to-step variability and metabolic cost of transport during human walking. *J. Exp. Biol.* 221, jeb181834. 10.1242/jeb.181834 30237239 PMC6262764

[JEB248125C155] Rodman, P. S. and McHenry, H. M. (1980). Bioenergetics and the origin of hominid bipedalism. *Am. J. Phys. Anthropol* 52, 103-106. 10.1002/ajpa.1330520113 6768300

[JEB248125C156] Roemmich, R. T., Leech, K. A., Gonzalez, A. J. and Bastian, A. J. (2019). Trading symmetry for energy cost during walking in healthy adults and persons poststroke. *Neurorehabil. Neural Repair* 33, 602-613. 10.1177/1545968319855028 31208276 PMC6688943

[JEB248125C157] Rotstein, A., Inbar, O., Berginsky, T. and Meckel, Y. (2005). Preferred transition speed between walking and running: effects of training status. *Med. Sci. Sports Exerc* 37, 1864-1870. 10.1249/01.mss.0000177217.12977.2f 16286854

[JEB248125C158] Rotto, D. M. and Kaufman, M. P. (1988). Effect of metabolic products of muscular contraction on discharge of group III and IV afferents. *J. Appl. Physiol. (1985)* 64, 2306-2313. 10.1152/jappl.1988.64.6.2306 3136123

[JEB248125C159] Rotto, D. M., Stebbins, C. L. and Kaufman, M. P. (1989). Reflex cardiovascular and ventilatory responses to increasing H+ activity in cat hindlimb muscle. *J. Appl. Physiol. (1985)* 67, 256-263. 10.1152/jappl.1989.67.1.256 2759951

[JEB248125C160] Rybicki, K. J., Waldrop, T. G. and Kaufman, M. P. (1985). Increasing gracilis muscle interstitial potassium concentrations stimulate group III and IV afferents. *J. Appl. Physiol. (1985)* 58, 936-941. 10.1152/jappl.1985.58.3.936 2984167

[JEB248125C161] Sanchez, N., Park, S. and Finley, J. M. (2017). Evidence of energetic optimization during adaptation differs for metabolic, mechanical, and perceptual estimates of energetic cost. *Sci. Rep.* 7, 7682. 10.1038/s41598-017-08147-y 28794494 PMC5550492

[JEB248125C162] Sanchez, N., Simha, S. N., Donelan, J. M. and Finley, J. M. (2019). Taking advantage of external mechanical work to reduce metabolic cost: the mechanics and energetics of split-belt treadmill walking. *J. Physiol. (Lond)* 597, 4053-4068. 10.1113/JP277725 31192458 PMC6675650

[JEB248125C163] Sanderson, D. J. (1991). The influence of cadence and power output on the biomechanics of force application during steady-rate cycling in competitive and recreational cyclists. *J. Sports Sci.* 9, 191-203. 10.1080/02640419108729880 1895355

[JEB248125C164] Sawicki, G. S., Beck, O. N., Kang, I. and Young, A. J. (2020). The exoskeleton expansion: improving walking and running economy. *J. Neuroeng. Rehabil* 17, 25. 10.1186/s12984-020-00663-9 32075669 PMC7029455

[JEB248125C165] Schrack, J. A., Simonsick, E. M., Chaves, P. H. and Ferrucci, L. (2012). The role of energetic cost in the age-related slowing of gait speed. *J. Am. Geriatr. Soc.* 60, 1811-1816. 10.1111/j.1532-5415.2012.04153.x 23035640 PMC3470763

[JEB248125C166] Seethapathi, N. and Srinivasan, M. (2015). The metabolic cost of changing walking speeds is significant, implies lower optimal speeds for shorter distances, and increases daily energy estimates. *Biol. Lett.* 11, 20150486. 10.1098/rsbl.2015.0486 26382072 PMC4614425

[JEB248125C167] Selinger, J. C. and Donelan, J. M. (2014). Estimating instantaneous energetic cost during non-steady-state gait. *J. Appl. Physiol.* 117, 1406-1415. 10.1152/japplphysiol.00445.2014 25257873

[JEB248125C168] Selinger, J. C. and Donelan, J. M. (2019). Behavioural energetics - An introduction. *International Society of Biomechanics*. Calgary, Alberta, Canada.

[JEB248125C169] Selinger, J. C., Hicks, J. L., Jackson, R. W., Wall-Scheffler, C. M., Chang, D. and Delp, S. L. (2022). Running in the wild: Energetics explain ecological running speeds. *Curr. Biol.* 32, 2309-2315.e3. 10.1016/j.cub.2022.03.076 35487220 PMC9169516

[JEB248125C170] Selinger, J. C., O'Connor, S. M., Wong, J. D. and Donelan, J. M. (2015). Humans can continuously optimize energetic cost during walking. *Curr. Biol.* 25, 2452-2456. 10.1016/j.cub.2015.08.016 26365256

[JEB248125C171] Selinger, J. C., Wong, J. D., Simha, S. N. and Donelan, J. M. (2019). How humans initiate energy optimization and converge on their optimal gaits. *J. Exp. Biol.* 222, 1-13. 10.1242/jeb.198234 31488623

[JEB248125C172] Shastri, L., Alkhalil, M., Forbes, C., El-Wadi, T., Rafferty, G., Ishida, K. and Formenti, F. (2019). Skeletal muscle oxygenation during cycling at different power output and cadence. *Physiol. Rep.* 7, e13963. 10.14814/phy2.13963 30734533 PMC6367161

[JEB248125C173] Sheehan, R. C. and Gottschall, J. S. (2013). Preferred step frequency during downhill running may be determined by muscle activity. *J. Electromyogr. Kinesiol* 23, 826-830. 10.1016/j.jelekin.2013.03.013 23628623

[JEB248125C174] Simha, S. N., Wong, J. D., Selinger, J. C., Abram, S. J. and Donelan, J. M. (2021). Increasing the gradient of energetic cost does not initiate adaptation in human walking. *J. Neurophysiol.* 126, 440-450. 10.1152/jn.00311.2020 34161744

[JEB248125C175] Simpson, C. S., Welker, C. G., Uhlrich, S. D., Sketch, S. M., Jackson, R. W., Delp, S. L., Collins, S. H., Selinger, J. C. and Hawkes, E. W. (2019). Connecting the legs with a spring improves human running economy. *J. Exp. Biol.* 222, jeb202895. 10.1242/jeb.202895 31395676 PMC6765174

[JEB248125C176] Skinner, N. E., Zelik, K. E. and Kuo, A. D. (2015). Subjective valuation of cushioning in a human drop landing task as quantified by trade-offs in mechanical work. *J. Biomech.* 48, 1887-1892. 10.1016/j.jbiomech.2015.04.029 25979381 PMC4492864

[JEB248125C177] Slade, P., Kochenderfer, M. J., Delp, S. L. and Collins, S. H. (2021). Sensing leg movement enhances wearable monitoring of energy expenditure. *Nat. Commun.* 12, 4312. 10.1038/s41467-021-24173-x 34257310 PMC8277831

[JEB248125C178] Smith, C. A., Rodman, J. R., Chenuel, B. J., Henderson, K. S. and Dempsey, J. A. (2006). Response time and sensitivity of the ventilatory response to CO2 in unanesthetized intact dogs: central vs. peripheral chemoreceptors. *J. Appl. Physiol. (1985)* 100, 13-19. 10.1152/japplphysiol.00926.2005 16166236

[JEB248125C179] Snyder, K. L. and Farley, C. T. (2011). Energetically optimal stride frequency in running: the effects of incline and decline. *J. Exp. Biol.* 214, 2089-2095. 10.1242/jeb.053157 21613526

[JEB248125C180] Snyder, K. L., Snaterse, M. and Donelan, J. M. (2012). Running perturbations reveal general strategies for step frequency selection. *J. Appl. Physiol. (1985)* 112, 1239-1247. 10.1152/japplphysiol.01156.2011 22241053

[JEB248125C181] Sockol, M. D., Raichlen, D. A. and Pontzer, H. (2007). Chimpanzee locomotor energetics and the origin of human bipedalism. *Proc. Natl. Acad. Sci. U.S.A* 104, 12265-12269. 10.1073/pnas.0703267104 17636134 PMC1941460

[JEB248125C182] Srinivasan, M. (2009). Optimal speeds for walking and running, and walking on a moving walkway. *Chaos* 19, 026112. 10.1063/1.3141428 19566272

[JEB248125C183] Steudel-Numbers, K. L. and Wall-Scheffler, C. M. (2009). Optimal running speed and the evolution of hominin hunting strategies. *J. Hum. Evol.* 56, 355-360. 10.1016/j.jhevol.2008.11.002 19297009

[JEB248125C184] Sutton, R. S. and Barto, A. G. (2018). *Reinforcement Learning: An Introduction*: MIT Press.

[JEB248125C185] Swinnen, W., Mylle, I., Hoogkamer, W., De Groote, F. and Vanwanseele, B. (2021). Changing stride frequency alters average joint power and power distributions during ground contact and leg swing in running. *Med. Sci. Sports Exerc* 53, 2111-2118. 10.1249/MSS.0000000000002692 33935233

[JEB248125C186] Tesio, L., Roi, G. S. and Möller, F. (1991). Pathological gaits: inefficiency is not a rule. *Clin. Biomech. (Bristol, Avon)* 6, 47-50. 10.1016/0268-0033(91)90041-N 23916344

[JEB248125C187] Umberger, B. R. and Martin, P. E. (2007). Mechanical power and efficiency of level walking with different stride rates. *J. Exp. Biol.* 210, 3255-3265. 10.1242/jeb.000950 17766303

[JEB248125C188] Van Caekenberghe, I., De Smet, K., Segers, V. and De Clercq, D. (2010). Overground vs. treadmill walk-to-run transition. *Gait Posture* 31, 420-428. 10.1016/j.gaitpost.2010.01.011 20219374

[JEB248125C189] van der Zee, T. J. and Kuo, A. D. (2021). The high energetic cost of rapid force development in muscle. *J. Exp. Biol.* 224, jeb233965. 10.1242/jeb.233965 33707194

[JEB248125C190] Van Hooren, B., Jukic, I., Cox, M., Frenken, K. G., Bautista, I. and Moore, I. S. (2024). The relationship between running biomechanics and running economy: a systematic review and meta-analysis of observational studies. *Sports Med.* 54, 1269-1316. 10.1007/s40279-024-01997-3 38446400 PMC11127892

[JEB248125C191] Walden, T. P., Fairchild, T., Girard, O., Peiffer, J. J., Jonson, A. M. and Dempsey, A. R. (2023). Blood flow restricted walking alters gait kinematics. *Eur. J. Sport Sci.* 23, 1528-1537. 10.1080/17461391.2023.2194274 36946174

[JEB248125C192] Warren, W. H. (1984). Perceiving affordances: Visual guidance of stair climbing. *J. Exp. Psychol. Hum. Percept. Perform* 10, 683-703. 10.1037/0096-1523.10.5.683 6238127

[JEB248125C193] Wasserman, K., Whipp, B. J., Koyal, S. N. and Cleary, M. G. (1975). Effect of carotid body resection on ventilatory and acid-base control during exercise. *J. Appl. Physiol. Respir. Environ. Exerc. Physiol.* 39, 354-358. 10.1152/jappl.1975.39.3.354 240799

[JEB248125C194] Weimerskirch, H., Martin, J., Clerquin, Y., Alexandre, P. and Jiraskova, S. (2001). Energy saving in flight formation. *Nature* 413, 697-698. 10.1038/35099670 11607019

[JEB248125C195] Whipp, B. J. and Ward, S. A. (1998). Determinants and control of breathing during muscular exercise. *Br. J. Sports Med.* 32, 199-211. 10.1136/bjsm.32.3.199 9773167 PMC1756098

[JEB248125C196] Wickler, S. J., Hoyt, D. F., Cogger, E. A. and Hall, K. M. (2001). Effect of load on preferred speed and cost of transport. *J. Appl. Physiol. (1985)* 90, 1548-1551. 10.1152/jappl.2001.90.4.1548 11247958

[JEB248125C197] Willcockson, M. A. and Wall-Scheffler, C. M. (2012). Reconsidering the effects of respiratory constraints on the optimal running speed. *Med. Sci. Sports Exerc* 44, 1344-1350. 10.1249/MSS.0b013e318248d907 22217570

[JEB248125C198] Windhorst, U. (2007). Muscle proprioceptive feedback and spinal networks. *Brain Res. Bull* 73, 155-202. 10.1016/j.brainresbull.2007.03.010 17562384

[JEB248125C199] Wolpert, D. M., Diedrichsen, J. and Flanagan, J. R. (2011). Principles of sensorimotor learning. *Nat. Rev. Neurosci.* 12, 739-751. 10.1038/nrn3112 22033537

[JEB248125C200] Wong, J. D., Cluff, T. and Kuo, A. D. (2021). The energetic basis for smooth human arm movements. *eLife* 10, e68013. 10.7554/eLife.68013 34927584 PMC8741215

[JEB248125C201] Wong, J. D., O'Connor, S. M., Selinger, J. C. and Donelan, J. M. (2017). Contribution of blood oxygen and carbon dioxide sensing to the energetic optimization of human walking. *J. Neurophysiol* 118, 1425-1433. 10.1152/jn.00195.2017 28637813 PMC5558034

[JEB248125C202] Wong, J. D., Selinger, J. C. and Donelan, J. M. (2019). Is natural variability in gait sufficient to initiate spontaneous energy optimization in human walking? *J. Neurophysiol* 121, 1848-1855. 10.1152/jn.00417.2018 30864867 PMC6589705

[JEB248125C203] Wulf, G. (2013). Attentional focus and motor learning: a review of 15 years. *Int. Rev. Sport Exerc. Psychol.* 6, 77-104. 10.1080/1750984X.2012.723728

[JEB248125C204] Yandell, M. B. and Zelik, K. E. (2016). Preferred barefoot step frequency is influenced by factors beyond minimizing metabolic rate. *Sci. Rep.* 6, 23243. 10.1038/srep23243 26988124 PMC4796793

[JEB248125C205] Zamparo, P., Francescato, M. P., De Luca, G., Lovati, L. and di Prampero, P. E. (1995). The energy cost of level walking in patients with hemiplegia. *Scand. J. Med. Sci. Sports* 5, 348-352. 10.1111/j.1600-0838.1995.tb00057.x 8775719

[JEB248125C206] Zarrugh, M. Y., Todd, F. N. and Ralston, H. J. (1974). Optimization of energy expenditure during level walking. *Eur. J. Appl. Physiol.* 33, 293-306. 10.1007/BF00430237 4442409

[JEB248125C207] Zhang, J., Fiers, P., Witte, K. A., Jackson, R. W., Poggensee, K. L., Atkeson, C. G. and Collins, S. H. (2017). Human-in-the-loop optimization of exoskeleton assistance during walking. *Science* 356, 1280-1284. 10.1126/science.aal5054 28642437

[JEB248125C208] Ziv, G. and Rotstein, A. (2009). Physiological characteristics of the preferred transition speed in racewalkers. *Med. Sci. Sports Exerc.* 41, 797-804. 10.1249/MSS.0b013e31818ff715 19276854

