# Supplementary material for: Behavioural energetics in human locomotion: how energy use influences how we move
Source: J Exp Biol. 2025 Feb 20;228(Suppl 1):JEB248125. doi: 10.1242/jeb.248125 (PMC11993254; doi:10.1242/jeb.248125)
Supplement: Supplementary information [file jexbio-228-248125-s1.pdf]

Supplementary Materials and Methods

Here we provide additional figure generation details for the figures displayed in the main text.

Fig. 1. For this figure, we chose to use data from pioneering studies if available and appropriate, otherwise we chose to use data that well represented the relationship as reported more broadly in the literature. In cases where data were available (via tables or text), we extracted the numbers directly (Abe et al., 2019). Otherwise, we extracted data from figures using the GRABIT function in MATLAB (R2022a) (Arellano and Kram, 2011; Donelan et al., 2001; Marsh and Martin, 1993; Ralston, 1958; Rathkey and Wall-Scheffler, 2017; Swinnen et al., 2021; Umberger and Martin, 2007). To create cost landscapes, we fit the data with polynomials of the same order as that reported in the original article when available (2nd order for Abe et al., 2019; Donelan et al., 2001; Marsh and Martin, 1993; Ralston, 1958; Rathkey and Wall-Scheffler, 2017; 3rd order for Umberger and Martin, 2007). In cases where data were not fit or fit parameters were not provided, we used a 2nd order polynomial (Arellano & Kram, 2011 and Swinnen et al., 2021). In studies that reported *net* metabolic power (Arellano and Kram, 2011; Donelan et al., 2001; Umberger and Martin, 2007), we added a resting cost of 1.22 W/kg to convert to gross metabolic power for comparison across studies (Glass et al., 2007; Long and Srinivasan, 2013). The walking and running speeds from experiments in **B** and **C** were similar to each other and to the energy optimal speeds reported in **A** (walking: 1.3m/s (Umberger and Martin, 2007)1.25m/s (Donelan et al., 2001); running: 3.3m/s (Swinnen et al., 2021) and 3m/s for (Arellano and Kram, 2011)). Participant numbers for each study are as follows: Ralston (1958) *(n=1), Abe et al. (2019)(n=13), Rathkey and Wall-Scheffler (2017) (n=9), Umberger and Martin (2007) (n=10), Swinnen et al. (2021) (n=17), Marsh and Martin (1993) (n=8), Donelan et al. (2001) (n=10), Arellano and Kram (2011) (n=11).*

Fig. 2. A: We combined and adapted Long and Srinivasan (2013) Figures 1a and 4c (n=28). We removed the individual participant data points and some labels from Figure 1a and removed the shading and labels from Figure 4c. **B:** We adapted Brown and Srinivasan (2021) Figure 2B (n=9). We removed the individual participant data points, the straight-line data, and the shaded curves representing 1 and 2% deviations from energy optima. We also used error bars to present the 25-75th percentiles instead of the full range of walking speeds for each radius. **C:** We adapted Daniels and Burn (2023) Figure 5a (n=13). We trimmed the x-axis limits to be within the range of data presented to limit white space in the figure.

Fig. 3. A: We reproduced Selinger et al. (2015) Figure 1A. **B:** We reproduced Selinger et al. (2015) Figure 3A (n=9). **C:** We adapted Roemmich et al. (2019) Figure 1A (to match the style of A). **D:** We combined and adapted Roemmich et al. (2019) Figures 3E, F (n=12). We converted the bar graph from 3E to data points using the GRABIT function in MATLAB (R2022a), keeping the error bars for the standard error of the mean. We fitted a second order polynomial curve to these data points for illustrative purposes.

Fig. 4. B: We scaled the latency of each sensor based on the following best-estimate values: vision = - 2s (prediction before movement initiation), motor command = -0.01s (in advance of movement if efference copy), proprioceptors=+0.01s, mechanosensitive group III = +0.03-0.2s and metabolite-sensitive group IV muscle afferents = 5-30s, chemoreceptors = 10-20s. **C:** We adapted O'Connor and Donelan (2012) Figure 1, removing aspects that were specific to their experiment and adapting the labels within the diagram to fit the language used in this review.

Fig. 5. A, B: We adapted Simpson et al. (2019a) Figures 5A and 2, making minor changes to the style for consistency with other figures in this review (n=4).

Journal of Experimental Biology • Supplementary information
